# Microbiota-Driven Metabolic Alterations Induced by BPA, TDCPP and PFOA in an Ex Vivo Human Fecal Fermentation Model

**DOI:** 10.1021/acs.chemrestox.5c00516

**Published:** 2026-03-17

**Authors:** Oscar Sabuz, Jacob Folz, Deepika Deepika, Jordi Blanco, Marta Schuhmacher, Georg Aichinger, Vikas Kumar

**Affiliations:** † Environmental Engineering Laboratory, TecnATox group, Departament d’Enginyeria Quimica, 16777 Universitat Rovira i Virgili, Av. Països Catalans 26, 43007 Tarragona, Catalonia, Spain; ‡ Laboratory of Toxicology and Environmental Health, Research in Neurobehavior and Health (NEUROLAB), School of Medicine, IISPV, Universitat Rovira i Virgili, 43201 Reus, Catalonia, Spain; § Pere Virgili Health Research Institute (IISPV), Department of Chemical Engineering, Universitat Rovira I Virgili, 43007 Tarragona, Spain; ∥ German Federal Institute for Risk Assessment (BfR), 10589 Berlin, Germany; ⊥ Laboratory of Toxicology, Department of Health Sciences and Technology, ETH Zürich, 8092 Zurich, Switzerland

## Abstract

The gut microbiome is increasingly recognized as a key contributor to chemical toxicity. Endocrine-disrupting chemicals (EDCs) such as bisphenol A (BPA), tris­(1,3-dichloro-2-propyl) phosphate (TDCPP), and perfluorooctanoic acid (PFOA) are widespread environmental contaminants with the potential to affect host health. To characterize microbiota-specific response to these compounds, we employed an ex vivo fecal fermentation model using samples from healthy adult donors. Fecal slurries were exposed to BPA, TDCPP and PFOA (75 μM) for up to 24 h under anaerobic conditions. Targeted LC–MS/MS quantified parent compounds over time, while untargeted metabolomics profiled microbial metabolic alterations at 4 and 24 h. TDCPP levels decreased similarly in fecal and abiotic controls, suggesting a nonmicrobial loss (e.g., instability or adsorption), whereas PFOA levels remained stable across donors. Untargeted metabolomics revealed compound- and time-dependent perturbations, with PFOA eliciting the strongest metabolic shifts. A curated set of 124 annotated metabolites indicated disruptions in bile acid transformation short-chain fatty acid production, nucleotide turnover, redox balance, and phytochemical catabolism. Several altered metabolites have been previously linked to immunomodulatory processes, suggesting potential implications for host–microbiota interactions. Overall, this study demonstrates the utility of ex vivo fermentation systems for assessing microbiota-mediated metabolic responses to xenobiotics and highlights the relevance of incorporating microbiome-related end points into chemical risk assessment.

## Graphical abstract

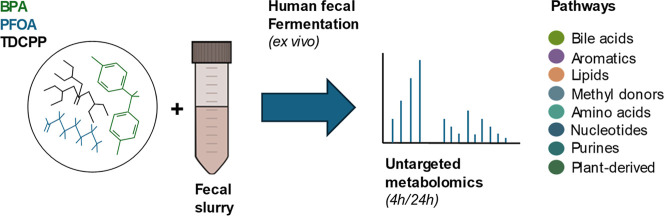

## 1 Introduction

Endocrine disrupting chemicals (EDCs) are a diverse group of environmental pollutants known to interfere with hormonal regulation and contribute adverse health outcomes across endocrine, reproductive, metabolic and immune systems.^1–4^ Human exposure to EDCs occurs through multiple routes—ingestion, inhalation, and dermal absorption—resulting in widespread internalization.^5–7^ Following ingestion, many EDCs reach the gastrointestinal tract and may directly interact with the gut microbiota, a community increasingly recognized as a determinant of xenobiotic fate and host physiology, including immune regulation.^2,7–9^

The human gut microbiota contributes to nutrient processing, immune homeostasis, and protection against pathogens,^10,11^ but also acts as a metabolic interface capable of transforming xenobiotics intro products of altered activity or toxicity.^12^ Conversely, chemical exposures—including EDCs—can perturb the microbial community structure and function.^13^ Such bidirectional interactions imply that microbiota metabolism may modulate both the bioavailability and biological impact of environmental contaminants, while microbial dysbiosis induced by pollutants has been linked to metabolic, inflammatory, and immune-mediated disorders.^14–17^ These insights have prompted a broader shift toward microbiome-aware toxicology and recognition that the gut microbiota can shape chemical dose–responses relationships and toxicity mechanisms.^18–22^

Among EDCs, bisphenol A (BPA), tris­(1,3-dichloro-propyl) phosphate (TDCPP) and perfluorooctanoic acid (PFOA) are of particular interest due to their high prevalence in human exposure and their reported effects on gut microbial ecology. Importantly, BPA and PFOA are among the best-characterized EDCs, with multiple studies—and recent evaluations by the European Food Safety Authority (EFSA)—highlighting their potential to modulate immune function in addition to endocrine and metabolic pathways.^23,24^ In contrast, TDCPP has received comparatively less attention, despite increasing detection in human biomonitoring studies and growing concern regarding its potential impacts on microbiota-associated and immune-related end points.^25,26^ BPA, a widely used plasticizer, has been extensively studied and it is known to alter gut microbial diversity, shift the abundance of key bacterial taxa and modulate short-chain fatty acid (SCFA) production in animal models. These microbial changes are often accompanied by systemic immune alterations, including shifts in cytokine responses and inflammatory tone.^27,28^ PFOA, a representative per- and polyfluoroalkyl substance (PFAS), is environmentally persistent and bioaccumulative, and has been linked to dysbiosis, altered lipid-metabolizing taxa and inflammatory signatures in animal models.^29,30^ EFSA has classified PFOA as immunotoxic based on human epidemiological and animal data, although direct evidence of microbiome-mediated immunotoxicity remains limited.^24,31^ By contrast, TDCPP—a widely used organophosphate flame retardant—has a far smaller evidence base. Existing studies indicate that TDCPP can disrupt microbial community composition in vitro and in vivo, decreasing microbial diversity and increasing potentially pathogenic taxa.^32,33^ These microbiota changes have been associated with heighted susceptibility to infection and immune dysfunction,^33–35^ although mechanistic understanding remains incomplete. Together, these three chemicals represent a relevant combination of well-characterized immunotoxic substances (BPA, PFOA) and a less-studied emerging flame retardant (TDCPP) for which microbiota-mediated effects are only beginning to be elucidated. Although existing studies collectively indicate that BPA, TDCPP and PFOA can influence gut microbiota structure and function, the specific microbial metabolic pathways disrupted by these exposures—and their potential relevance to host physiology—remain poorly defined.

Despite the recognized role of the microbiome as a mediator of chemical toxicity, most toxicological testing frameworks still rely on in vitro monocultures or animal models that do not explicitly account for gut microbial contributions.^18,21,36,37^ Microbiota-specific response, microbial metabolites, and microbiota-mediated modes of action are rarely captured using these approaches, contributing to gaps in mechanistic understanding and limiting the integration of microbial end points into risk assessment.^38,39^ Increasing evidence suggests that certain toxicological outcomes—including immune perturbation—may arise not solely form host responses to parent compounds, but from microbially mediated transformations or metabolic disruptions.^40–42^

Ex vivo fecal fermentation models provide a useful complementary approach for characterizing microbiota-specific response to xenobiotics under controlled, anaerobic conditions, without the confounding influence of host physiology.^43–45^ The 96-deepwell plate format used in this study offers a high-throughput platform that enables direct comparison of microbial metabolic responses across compounds, donors, and time points.^46^ Although these systems do not fully recapitulate the spatial and physicochemical complexity of the human gastrointestinal achievable with larger anaerobic bioreactors, they yield producible community-level responses and are increasingly adopted as screening tools for microbiota–chemical interactions.^45–47^

With this rationale, the aim of this study was to investigate whether BPA, TDCPP and PFOA undergo microbially mediated metabolism in an ex vivo human fecal fermentation model, and to characterize the compound- and time-dependent effects of these exposures on microbial metabolic activity. By applying untargeted LC–MS/MS-based metabolomics integrated with multivariate analyses, we sought to identify key metabolite pathways affected by each xenobiotic and explore whether the altered metabolites included compounds with previously described immunomodulatory functions. This approach provides insight into early indicators of xenobiotic-induced dysbiosis and identifies candidate microbial metabolites that may contribute to downstream host responses.

## 2 Methods

### 2.1 Chemicals and Stocks Solutions

Bisphenol A (BPA), perfluorooctanoic acid (PFOA) and tris­(1,3-dichloro-2-propyl) phosphate (TDCPP), were purchased from Sigma-Aldrich (Darmstadt, Germany). Stock solutions of 600 mM were prepared in dimethyl sulfoxide (DMSO) and subsequently diluted to the desired working concentrations.

### 2.2 Fecal Sample Collection

This study was exempted from review by the Cantonal Ethics Commission of Zurich. Fresh fecal samples were collected from five healthy adult donors (1 female and 4 males), and in an anonymized, noninterventional manner, following written informed consent and confirmation of inclusion criteria compliance. Eligible donors were required to have no history of inflammatory bowel disease or other chronic gastrointestinal disorders, and to have not taken antibiotics within the previous 30 days, ensuring a stable and representative gut microbiota. Samples were self-collected using sterile plastic containers containing an Oxoid AnaeroGen pouches (Thermo Fisher Diagnostics AG, Pratteln, Switzerland), to induce anaerobiosis. All samples were processed within 3 h of collection inside an anaerobic chamber. To prepare a 20% (w/v) fecal slurry (FS), 8g of fecal matter homogenized with 40 mL of anaerobic phosphate buffer (PBS) containing glycerol 15% (v/v) using glass beads and a vortex. The mixture was filtered and stored at −80 °C. A pooled FS sample was prepared by combining 10 mL of each individual FS.

### 2.3 Microbiota Culture Medium

Fecal fermentation experiments were conducted using bYCFA medium supplemented with a heat-stable 6C + Muc component, a complex carbohydrate and mucin-rich mixture designed to mimic the nutrient composition of the human colon and support the growth of a diverse gut microbiota.^46^ The preparation included amicase (Sigma-Aldrich Chemie GmbH, Buchs, Switzerland), yeast extract (Lesaffre, Marcq-en-Barœul, France), meat extract (Sigma-Aldrich), mineral solution I (K_2_HPO_4_), mineral solution II (KH_2_PO_4_, NaCl, (NH_4_)_2_SO_4_, MgSO_4_, CaCl_2_), soluble starch (Sigma-Aldrich), pectin from citrus peel (Sigma-Aldrich), xylan (Angene, London, United Kingdom), arabinogalactan (Sigma-Aldrich), guar gum (Sigma-Aldrich), inulin (Beneo, Mannheim, Germany), mucin type II (Sigma-Aldrich), vitamin solution (biotin, cobalamin, 4-aminobenzoic acid, folic acid and pyridoxamine, Sigma-Aldrich), and volatile fatty acids solution (acetic acid (Sigma-Aldrich), propionic acid (Sigma-Aldrich), valeric acid (VWR International AG, Dietikon, Switzerland), isovaleric acid (Sigma-Aldrich), and isobutyric acid (Sigma-Aldrich) in 5 M NaOH). All components were dissolved in dH_2_O, adjusted to pH 7, and boiled for 10 min. After cooling to ∼60 °C, l-cysteine HCl and NaHCO_3_ were added under constant N_2_ flushing. The final mixture was transferred to DURAN Pressure flasks (DWK Life Sciences GmbH, Wertheim, Germany) and autoclaved.

### 2.4 Ex Vivo Fermentation and Exposure

Fermentation assays were performed in 96-deepwell plates (1.8 mL) under anaerobic conditions at 37 °C. FS 20% (w/v) were diluted with complete medium to achieve a final concentration of 5% (w/v) feces. For experiments evaluating xenobiotic stability (targeted analysis of TDCPP and PFOA), both individual FS samples and a pooled FS were included to assess potential interdonor variability and to distinguish microbiota from abiotic effects. In contrast, untargeted metabolomics was conducted exclusively on the pooled FS to minimize donor-specific variability and enhance the detection of community-level metabolic responses. All experimental conditions were performed in biological triplicates (*n* = 3), and all samples were processed in parallel under the same conditions. A control without FS (non-FS control) was also included to account for potential abiotic effects of the compounds in the absence of microbial metabolism. The medium was supplemented with BPA, TDCPP or PFOA at a final concentration of 75 μM, or 0.1% DMSO (control vehicle). This concentration was selected to ensure consistency with parallel in vitro experiments performed as part of this research project, allowing for cross-model comparisons between immune and microbiota-focused assays. Outer wells were filed with dH_2_O or PBS to reduce evaporation.^48^ Sampling was performed at 0, 4 and 24 h to capture both early and later microbial responses to xenobiotic exposure. Samples (20 μL) were transferred to a 96-well plate containing 180 μL of methanol 100% (v/v) with internal standard sulfamethoxazole (400 nM), quickly sealed and stored at −20 °C. Prior to LC–MS/MS analysis, plates were thawed on ice, centrifuged at 2204*g* for 30 min at 4 °C, and 80 μL of supernatant were transferred into a new MS-compatible plate for metabolomic analysis.

### 2.5 Standard Curve Preparation

Calibration curves for BPA, TDCPP, and PFOA were generated by preparing serial dilutions in methanol. Each standard solution was spiked with sulfamethoxazole as internal standard and analyzed under the same MS/MS conditions used for the experimental samples. The curves showed high linearity with correlation coefficients (*R* ^2^) > 0.95 for all compounds.

### 2.6 Mass Spectrometry Analysis

Chemicals analysis was performed using a Vanquish HPLC (ThermoFisher) linked with an IDX tribrid LC–MS/MS mass spectrometer (ThermoFisher). Resuspended samples were stored in the autosampler at 4 °C until analysis. Injection volume was 5 μL. A Synergi 30 mm × 2 mm (4 μm Polar-RP 80 Å) LC column (Phenomenex) was kept at 40 °C and had a constant flow rate of 0.7 mL per minute. Mobile phase A was LC–MS grade water with 0.1% formic acid, and mobile phase B was LC–MS grade acetonitrile with 0.1% formic acid. The LC gradient was 5% B from 0 to 0.1 min, brought to 100% B between 0.1 and 1.8 min, kept at 100% B until 2.35 min, brought to 5% B between 2.35 and 2.4 min, and kept at 5% B from 2.4 to 2.8 min. The autosampler needle wash and injection process took approximately 1 min between each sample. Ionization source conditions for sheath gas, aux gas, and sweep gas were 60, 15, and 2 respectively. The transfer tube was at 350 °C, and the vaporizer temperature was 400 °C. MS1 spectra were acquired at 60 k resolution between 60 and 800 *m*/*z* with RF lens of 45%, and maximum injection time of 50 ms with default AGC target. Targeted MS/MS scans were performed using the linear ion trap with fragmentation energy and RF lens % values optimized to each targeted metabolite. Both positive and negative ionization modes were employed on separate injections with +3.5 kV and −2.5 kV spray voltages. For identification of nontargeted LC–MS features, a pooled sample was analyzed 9 times with iterative exclusion data dependent MS/MS spectra acquired at 15 k resolving power in the orbital ion trap and the top two ions selected for fragmentation using 2.5 s dynamic exclusion.

### 2.7 Metabolomic Data Analysis

Targeted MS/MS analysis was used to quantify the concentrations of TDCPP (positive ionization mode) and PFOA (negative ionization mode) during fermentation using both individual and pooled FS samples. Raw data were corrected using the internal standard sulfamethoxazole, and final compound concentrations were determined at 0, 4 and 24 h by interpolation against a standard calibration curve. BPA was excluded from the targeted analysis due to unsuccessful detection under the tested conditions, despite multiple attempts using different ionization modes and acquisition settings. However, BPA was included in the untargeted metabolomic analysis. Untargeted metabolomics profiling was performed using LC–MS/MS from positive ionization mode analysis, yielding 6816 chromatographic features. Metabolite annotation was performed using MS/MS spectra from 10 iterative exclusion acquired DDA scans performed on the pooled FS sample matched to a MS/MS spectral library consisting of NIST23 and the MassBank of North America (MoNA). Only annotations from features of statistical interest are reported. Features annotated as unknowns, lacking MS/MS confirmation, or duplicate entries were excluded. This filtering process resulted in a curated list of 124 annotated metabolites, which were subsequently used in univariate and multivariate analyses. Untargeted metabolomics were performed on pooled FS samples exposed to 75 μM of each EDC at 4 h and 24 h. This approach aimed to maximize the detection of metabolic shifts and identify relevant pathways under strong exposure conditions, while minimizing redundancy across dose levels. For univariate analysis, normalized intensity values were compared against DMSO-treated controls using student’s *t*-test, as implemented in MetaboAnalyst 5.0. Fold changes were calculated as the ratio between the mean intensity in the exposed group and the mean intensity in the control group. Volcano plots were generated by plotting—log_10_(*p*-value) against log_2_(fold change). A significance threshold of *p* < 0.05 (−log_10_(*p*) > 1.3) was applied. Although all calculations were performed in MetaboAnalyst, volcano plots were visualized in GraphPad Prism using the exported values for enhanced clarity. For multivariate analysis, including partial least-squares discriminant analysis (PLS-DA) and hierarchical clustering (heatmaps), the data set was normalized by sum and scaled using Pareto scaling to reduce the influence of large absolute differences while retaining relative variation across treatment groups. These analyses were also conducted in MetaboAnalyst 5.0 to visualize global metabolic shifts and treatment-specific clustering.

### 2.8 Statistical Analysis

Data analysis was performed using IBM SPSS Statistics, version 22.0 (IBM Corp., Armonk, NY, USA). One-way analysis of variance (ANOVA) was used to evaluate group differences. Where applicable, post hoc comparisons were conducted using Dunnett’s multiple comparison tests. Statistical significance was defined as *p < 0.05, **p < 0.01 and ***p < 0.001.

## 3 Results

### 3.1 Microbial Degradation of Environmental EDCs

To evaluate the stability and potential microbial transformation of environmentally relevant EDCs, targeted MS/MS analysis was employed to quantify the concentrations of TDCPP and PFOA over time during ex-vivo fermentation. Both individual and pooled FS samples were analyzed to assess interindividual differences in microbial degradation capacity.

TDCPP showed a time-dependent decreased concentration in both FS-containing and medium-only wells (Figure 1A–F), suggesting that the decline was not driven by microbial activity. In contrast, the concentration of PFOA increased over time in both conditions (Figure 1G–L). Considering PFOA’s chemical stability, this observation is most likely due to analytical variability or a potential artifact. Statistical analysis revealed no significant differences in compound concentrations between FS and non-FS conditions at any time point or donor, further supporting the absence of microbiota-mediated degradation.

**Figure 1:**
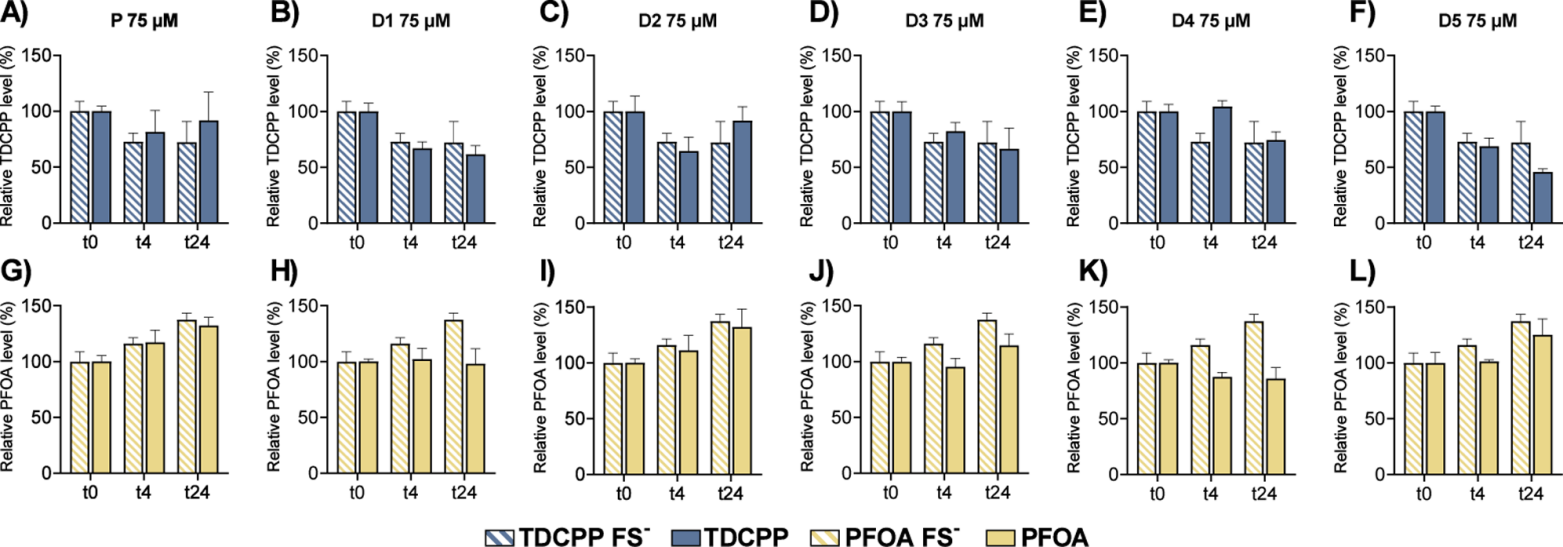
Targeted MS/MS quantification of EDCs in pooled and individual fecal slurry (FS) samples. The first row (A–F) shows TDCPP-exposed samples, and the second row (G–L) shows PFOA-exposed samples. For each compound, the graphs represent pooled (P) FS exposed to 75 μM, followed by individual FS donors (D1–5) exposed to 75 μM. Stripped bars represent non-FS controls. Data are presented as mean ± standard deviation (SD), *n* = 3. Statistical significance was determined using Dunnett’s multiple comparisons test: *p* < 0.05, *p* < 0.01, *p* < 0.001.

To further investigate possible TDCPP metabolism, a manual inspection of the untargeted metabolomics data set was conducted to search for known degradation products such as bis­(1,3-dichloro-2-propyl) phosphate (BDCPP). However, no candidate metabolites could be confidently detected based on MS/MS spectral matching or retention time criteria. Future studies should consider incorporating targeted MS/MS or suspect screening approaches to specifically track TDCPP transformation. Overall, the concentration profiles of TDCPP and PFOA over time were similar in both FS and no-FS conditions, suggesting that no substantial microbial degradation occurred under the tested conditions.

### 3.2 Alterations of Gut Microbial Metabolism

To assess the metabolic impact of chemical exposure on the gut microbiota, fecal slurry samples were incubated ex vivo with BPA, TDCPP, or PFOA at 75 μM for 4 and 24 h. Untargeted metabolomic profiling was used to identify metabolic features showing significant differences relative to DMSO-treated controls at each time point (Figure 2). Significance was defined using univariate *t* tests (*p* < 0.05) as an exploratory criterion to highlight candidate metabolites for downstream multivariate interpretation.

**Figure 2:**
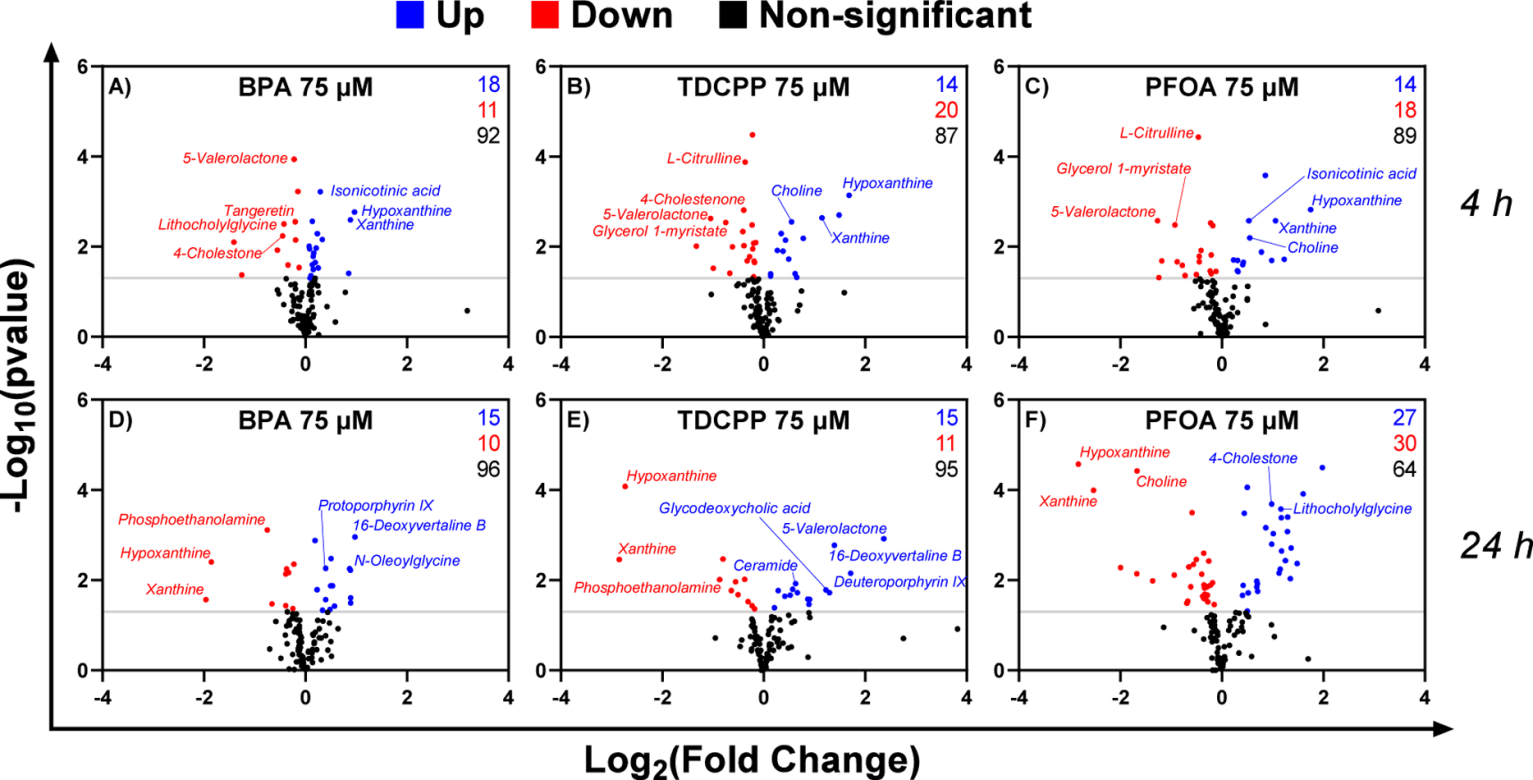
Volcano plots from the untargeted metabolomic analysis of pooled fecal slurry (FS) using LC–MS/MS. Fermentations were carried out for 4 h (A–C) and 24 h (D–F) in the presence of BPA, TDCPP, and PFOA 75 μM (*n* = 3). A total of 124 annotated metabolic features were included in the analysis. Univariate comparisons between DMSO-treated controls and EDC-exposed samples were performed using Student’s *t*-test. Fold changes were calculated as the ratio between the mean intensity in exposed samples and their corresponding controls and expressed as log_2_(fold change). Volcano plots display—log_10_(*p*-value) versus log_2_(fold change), with features meeting the exploratory significance threshold of *p* < 0.05 (−log_10_(*p*) > 1.3) considered significantly altered. Black dots represent nonsignificant features; blue dots indicate significantly increased metabolites in exposed samples; and red dots indicate significantly decreased metabolites. Selected annotated metabolites from the top five most upregulated or downregulated features are labeled in each panel.

Overall, PFOA induced the strongest metabolic shifts. At 24 h, 27 features were significantly upregulated and 30 were downregulated (Figure 2F), whereas at 4 h 14 features increased and 18 decreased (Figure 2C). BPA and TDCPP induced more moderate alterations. BPA exposure led to 29 and 25 altered features at 4 h (Figure 2A) and 24 h (Figure 2D), respectively, with a slight predominance of upregulated features at 4 h. TDCPP caused a similar number of changes at both time points (34 at 4 h and 26 at 24 h), but with a higher number of downregulated features at 4 h (Figure 2B,E). These results indicate that PFOA exerts a broader and more sustained impact on microbial metabolism compared to BPA and TDCPP.

All significant features, together with fold changes and *p*-values, are listed in Supplementary Table S1. Representative annotated metabolites are highlighted in Figure 2. For BPA, exposure at 4 h led to modest increase in purine metabolites such as hypoxanthine and xanthine, as well as isonicotinic acid, while compounds like 5-valerolactone, tangeritin and lithocholylglycine were slightly downregulated. At 24 h, the most downregulated metabolites included hypoxanthine and phospholipid derivates such as phosphoethanolamine, whereas upregulated metabolites included *N*-oleolglycine, 16-deoxyvertaline B, sphingosine and protoporphyrin IX. TDCPP exposure showed stronger alterations at 4 h, with significant upregulation of hypoxanthine, xanthine, and choline, and downregulation of 5-valerolactone, 4-cholestenone, 5-(carbamoylamino) pentanoic acid and glycerol 1-myristate. At 24 h, however, purine levels were markedly decreased and bile acids such as glycodeoxycholic acid were upregulated. PFOA exhibited the strongest and most consistent impact. At both time points, hypoxanthine and xanthine were significantly altered, with a switch from upregulation at 4 h to marked downregulation at 24 h. Other strongly affected metabolites included 5-valerolactone and choline, which showed opposite trends depending on time.

In summary, volcano plot analysis revealed compound- and time-specific metabolic alterations following exposure to BPA, TDCPP and PFOA at 4 h and 24 h. PFOA elicited the most extensive changes, particularly at 24 h, with the highest number of up- and down-regulated features. BPA and TDCPP showed more moderate effects, with TDCPP producing slightly more changes at 4 h. Several metabolites, including hypoxanthine, xanthine, choline, 5-valerolactone, and bile acid derivatives, were among the most consistently affected across compounds and time points. These findings provide a basis for subsequent multivariate analyses to determine broader pathway-level disruptions.

The PLS-DA model identified a set of metabolites with high variable importance in projection (VIP) scores, which significantly contributed to the separation between DMSO-treated controls and samples exposed to 75 μM of each EDC at 4 h and 24 h of ex vivo fermentation (Supplementary Figure S1). Functional classification of these VIP metabolites revealed consistent alterations in several key pathways associated with bile acid metabolism, lipid remodeling, purine degradation and microbial processing of dietary compounds.

At 4 h (Figure 3A), relevant VIPs included glycodeoxycholic acid and stercobilin (bile acid metabolism), as well as pentamethoxyflavone isonicotinic acid and tangeritin (aromatic and polyphenol catabolism). Several lipid-related metabolites—such as glycerol 1-myristate, palmitoyl sphingomyelin, phosphoetanolamine, and heptadecaphinganine—were also detected at his time point, together with 5-valerolactone (a SCFA-related intermediate), choline, and multiple dipeptides including Val–Pro, Pro–Phe, and proline-hydroxiproline, as well as 5-(carbamoylamino) pentanoic (l-citrulline). Additionally, heme derivatives (e.g., deuteroprophyrin IX, protoprophyrin IX) and purine degradation products (hypoxanthine, xanthine) appeared as relevant VIPs.

**Figure 3:**
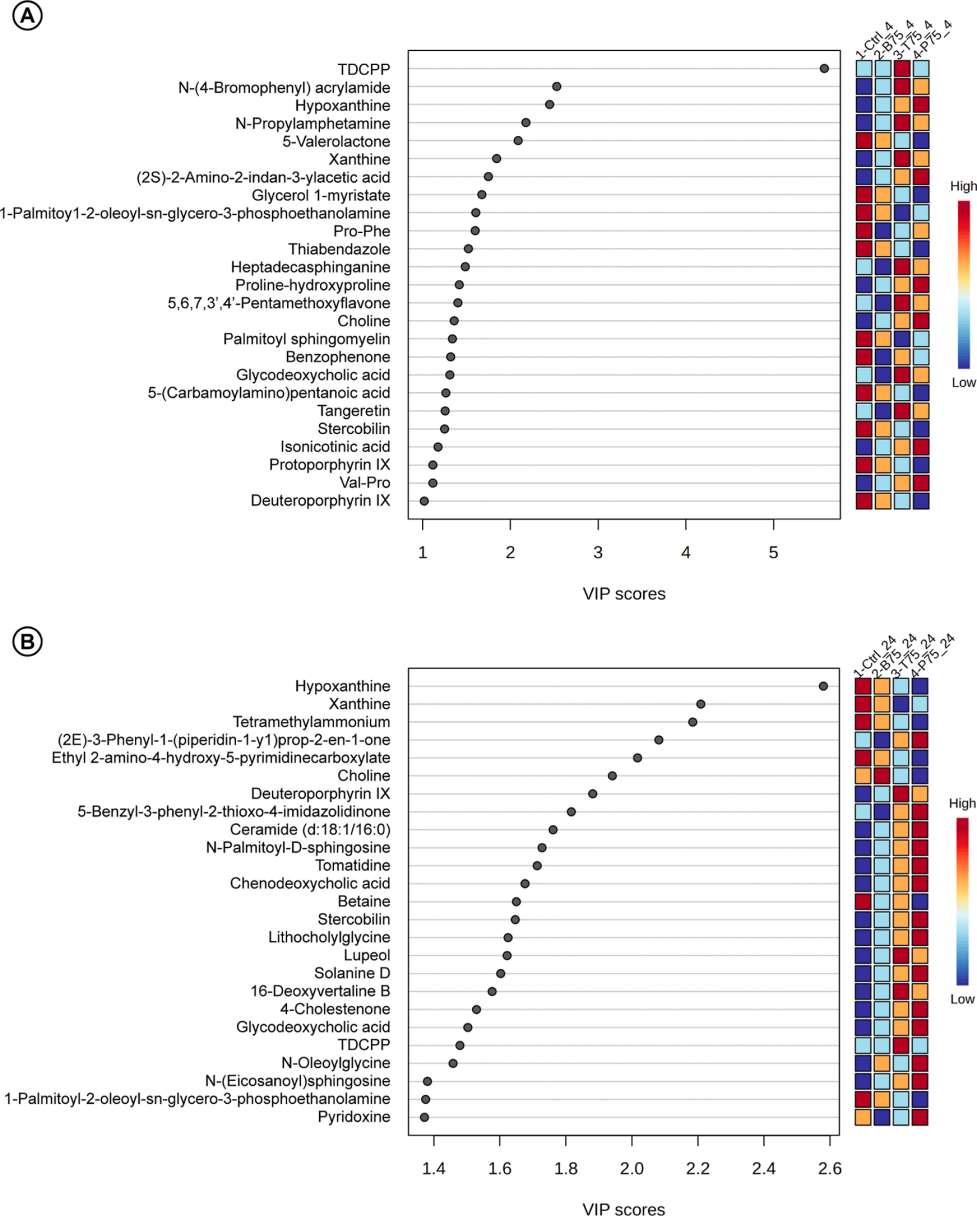
VIP score plots derived from PLS-DA analysis of fecal slurry samples exposed to 75 μM of BPA, TDCPP and PFOA for (A) 4 h and (B) 24 h. Models were constructed to discriminate between DMSO-treated controls and EDC-exposed groups based on untargeted metabolomic profiles. The plots show the top 25 metabolites ranked by variable importance in projection (VIP) scores, representing the features that contributed most strongly to group separation. Only annotated metabolites with MS/MS support are included.

At 24 h (Figure 3B), the VIP profile shifted to include bile acid derivatives such as chenodeoxycholic acid and lithocholylglycine, alongside increased levels of ceramide d18:1/16:0, *N*-palmitoyl-d-sphingosine, *N*-(eicosanoyl)­sphingosine, *N*-oleoglycine and phosphoetanolamine. Pyrimidine and plant-derived compounds including tomatidine, solanine D, lupeol, 16-deoxyvertaline B and pyridoxine were also identified exclusively at this later time point. Deuteroporphyrin IX and choline were retained as VIPs at 24 h, as well as betaine.

### 3.3 Time- and Compound Specific Impacts on Metabolomic Profiles

Building upon the identification of significantly altered metabolites, we further explored global metabolomic changes using multivariate analyses. Heatmaps and VIP scores derived from PLS-DA models revealed compound- and time-specific effects on microbial metabolism following EDC exposure. These analyses provide insight into overall metabolic patterns and identify key metabolites driving group separation. These observations were supported by normalized abundance values shown in Supplementary Table S2.

At 4 h (Figure 4A), early changes were observed in several key metabolic pathways. Bile acid metabolism was already affected. Glycodeoxycholic acid showed an increase with TDCPP and PFOA, while stercobilin was markedly reduced across all treatments. Polyphenol- and aromatic-derived metabolites increased at 4 h, particularly under TDCPP and PFOA exposure, but no longer showed significant changes at 24 h. Similarly, the SCFA-related intermediate 5-valerolactone was significantly decreased at 4 h in all EDC treatments and absent from VIP rankings at 24 h. Concerning lipid metabolism, several structural lipids were downregulated, including glycerol 1-myristate, phosphoethanolamines, and palmitoyl sphingomyelin, while heptadecaphinganine increased with TDCPP and PFOA. Regarding methyl donor pathways, choline was consistently increased, while peptide-related changes included Val–Pro, Pro–Phe, proline-hydroxyproline and l-citrulline alterations, with proline derivatives increasing and others decreasing. Purine metabolites such as hypoxanthine and xanthine were also elevated at 4 h across all treatments. Finally, both deuteroporphyrin IX and protoporphyrin IX showed early reduction at this time point.

**Figure 4:**
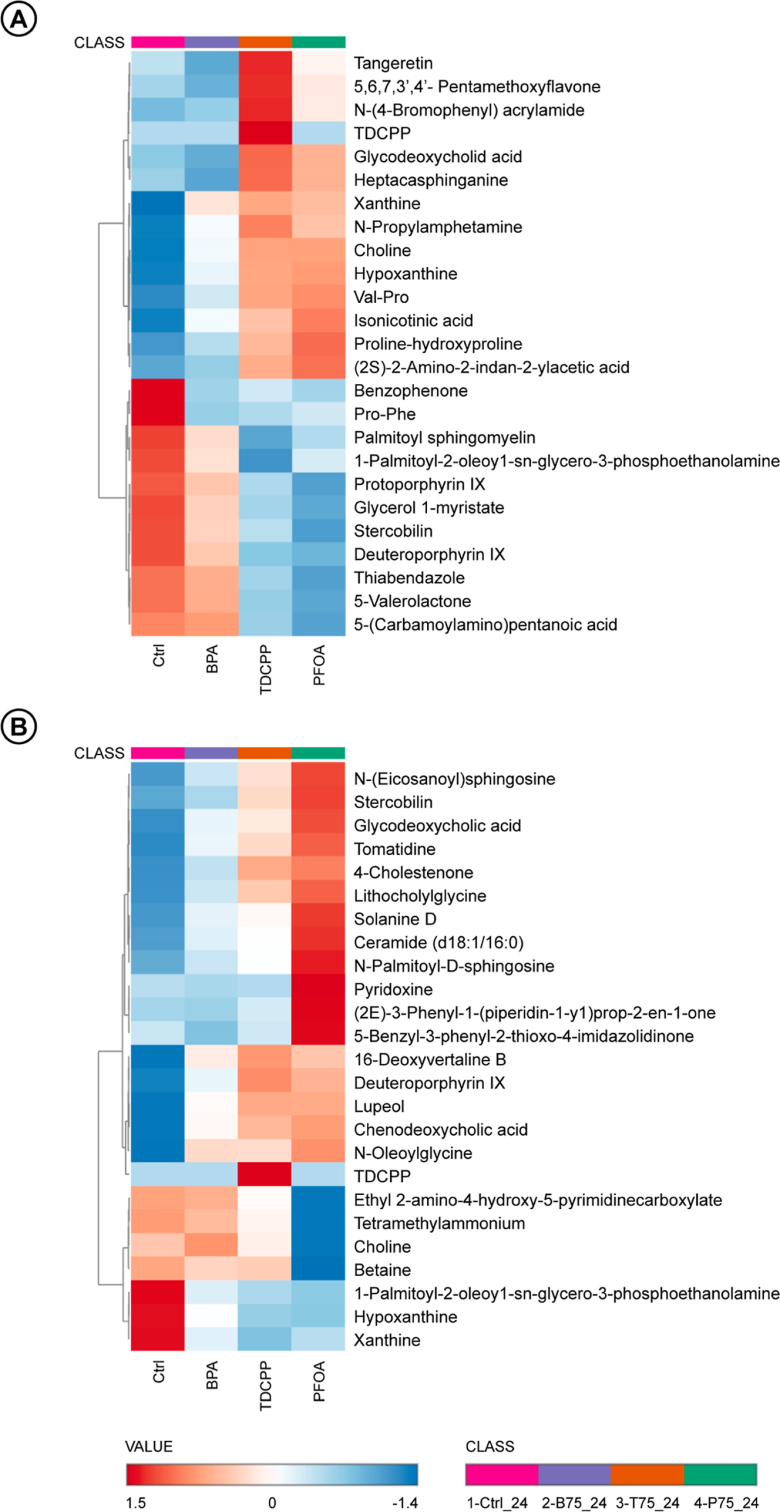
Heatmaps of normalized relative abundances for VIP metabolites selected by PLS-DA after exposure of fecal slurry samples to 75 μM of BPA, TDCPP or PFOA. Panels correspond to (A) 4 h and (B) 24 h of incubation. Each row represents an annotated VIP metabolite, and each column corresponds to treatment condition, including DMSO-treated controls. Color intensity reflects auto scaled values (mean-centered and divided by the standard deviation for each metabolite), illustrating relative increases or decreases across conditions.

At 24 h (Figure 4B), the metabolic alterations observed at 4 h became more extensive, affecting additional pathways and showing a different pattern of metabolite changes. Bile acid metabolism showed broader activation, with elevated levels of stercobilin, glycodeoxycholic acid, chenodeoxycholic acid and lithocholylglycine. Lipid metabolism displayed consistent upregulation of sphingolipids and ceramides, including ceramide d18:1/16:0, *N*-palmitoyl-d-sphingosine, *N*-(eicosanoyl) sphingosine and *N*-oleoylglycine, while phosphoethanolamine remained suppressed. In methyl donor metabolism, choline showed compound-specific responses, while betaine was consistently decreased, particularly under TDCPP and PFOA. In contrast whit 4 h, peptide-related metabolites no longer showed relevant differences at 24 h. Pyrimidine was consistently decreased while the heme-derivatives such as deuteroporphyrin IX were elevated. Hypoxanthine and xanthine showed a significant decrease at 24 h, in contrast to 4 h. Plant-derived compounds were consistently elevated across all EDC treatments.

Together, VIP analysis and heatmap profiling indicate that short-term and prolonged exposure to EDCs induces dynamic shifts in microbial metabolism. These effects are especially evident in pathways related to bile acid turnover, polyphenol catabolism, membrane lipid composition, amino acid metabolism, and redox-sensitive processes.

## 4 Discussion

Emerging evidence highlights gut microbiota as a key contributor to xenobiotic metabolism and a potential modulator of host response to environmental chemicals. In this study, we used an ex vivo fecal fermentation model to investigate how selected EDCs—namely BPA, TDCPP and PFOA—affect microbial metabolic activity over time. By integrating targeted quantification, untargeted metabolomics and multivariate analyses, we identified compound-specific and time-dependent alterations across multiple microbial pathways. Several metabolites affected by EDC exposure have been previously linked to immune regulation, suggesting potential—but untested—implications for host–microbiota interactions.

We first evaluated whether the selected EDCs were metabolized or accumulated over time in the fecal environment. Targeted MS/MS analysis revealed no evidence of microbially driven transformation of TDCPP or PFOA. TDCPP levels declined similarly in FS-containing and abiotic controls, suggesting physicochemical loss rather than biodegradation. PFOA remained stable or increased slightly across donor sand pooled samples, consistent with the well-documented analytical challenges and physicochemical persistence of fluorinated compounds.^49–52^ BPA was not detectable in the targeted workflow, likely due to ionization inefficiency or matrix-induced signal suppression, compound adsorption to plastic surfaces, or suboptimal instrument settings, as widely reported for complex biological matrices.^53–55^ These results emphasize the need for robust abiotic controls and compound-optimized methods when assessing xenobiotic metabolism in microbiota-based systems.

To gain a broader view of microbial metabolic disruption, we next explored untargeted metabolomics data using univariate and multivariate approaches. Volcano plot analysis provided a first-layer overview of significantly altered metabolic features following EDC exposure. The number and direction of dysregulated metabolites varied with compound and incubation time. While BPA and TDCPP induced modest changes, PFOA elicited the strongest and most sustained perturbations, particularly at 24 h, with over 50 features significantly upregulated or downregulated. This highlights a higher disruptive potential of PFOA on microbial metabolism, possibly due to differences in physicochemical properties. Several metabolites emerged as recurrent across exposure, including purine degradation products (hypoxanthine, xanthine), bile acid derivatives (e.g., lithocholylglycine), 5-valerolactone, and choline. Interestingly, the direction of change often shifted over time—for example, hypoxanthine and choline showed early upregulation followed by suppression at 24 h. These temporal patterns may reflect an initial phase of microbial stress response or nucleotide turnover, followed by adaptation or exhaustion of metabolic pathways.

Multivariate analysis provided deeper insight into the functional pathways disrupted by xenobiotic exposure. Across compounds and time points, several biochemical domains emerged as recurrently affected, reflecting a coordinated microbial response rather than isolated metabolites changes. Bile acid metabolism showed one of the clearest time-structured responses. Early reductions of stercobilin at 4 h were followed by the appearance or accumulation of secondary bile acid derivatives such as glycodeoxycholic acid, chenodeoxycholic acid and lithocholylglycine at 24 h. These patterns suggest a progressive disturbance of microbial bile salt deconjugation and transformation, consistent with prior observation that environmental toxicants can modulate bile acid-metabolizing communities and pathways.^56,57^ Aromatic and polyphenol-derived metabolites, such as pentamethoxyflavone and isonicotinic acid, were prominent VIP features at 4 h, but largely absent by 24 h. Because these compounds are typically processed by specialized microbial taxa, their transient enrichment may reflect early substrate–driven activity followed by depletion, shifts in the abundance of flavonoid-catabolizing bacteria, or altered enzymatic capacity under prolonged xenobiotic stress.^58–60^ SCFA-related pathways also appeared sensitive to chemical exposure. The consistent reduction of 5-valerolactone at 4 h points to transient impairment in microbial degradation of dietary fibers and flavan-3-ols. Its absence among 24 h VIP features suggest sustained alterations in SCFA-linked fermentation, aligning with literature showing that pollutants can reduce SCFA-producing taxa and compromise carbohydrate metabolism.^61–64^ Lipid remodeling exhibited a clear temporal bifurcation: glycerolipids and phospholipid precursors (e.g., phosphoethanolamine, glycerol 1-myristate) were reduced at 4 h, whereas ceramides, sphingomyelins and other sphingolipid derivatives accumulated at 24 h. Such patterns are characteristic of microbial adaptation to environmental stressors, potentially involving membrane restructuring or altered fatty acid fermentation.^65^ Compounds involved in one-carbon metabolism, notably choline and betaine, also showed compound- and time-dependent dynamics. Choline tended to increase at 4 h and decrease at 24 h, whereas betaine was consistently diminished across exposures. These fluctuations may reflect disruptions methyl-donor availability and potential microbial stress response.^66^ Several peptides and amino acid derivatives were differentially regulated at 4 h, consistent with enhanced proteolysis or altered amino acid fermentation, commonly associated with stress adaptation.^67^ Perturbations in nucleotide metabolism were also evident. Purine intermediates (hypoxanthine, xanthine) increased at 4 h but decreased markedly at 24 h, suggesting an early rise in nucleotide turnover or oxidative stress, followed by metabolic suppression or resource depletion. In parallel, decreased pyrimidines at 24 h indicate broader disruption of nucleic acid precursors. Such combined purine/pyrimidine alterations are commonly observed in microbiota exposed to chemical or oxidative stress.^68,69^ Porphyrin and heme-related metabolites displayed a similarly dynamic trajectory. Protoporphyrin IX and deuteroporphyrin IX were reduced early, yet accumulated at 24 h, consistent with alterations in microbial heme utilization, scavenging capacity or redox status.^70,71^ Their delayed increase may reflect microbial stress, changes in iron availability or shifts in taxa that either degrade or accumulate porphyrin derivatives.^72–74^ Finally, plant-derived and phytochemical metabolites showed some of the strongest late-stage responses. Tomatidine, solanine D, lupeol, 16-deoxyvertaline B and pyridoxine were at the top VIP features at 24 h, whereas tangeritin only ranked highly at 4 h. This transition likely reflects delayed engagement of microbial pathways responsible for phytochemical catabolism or changes in microbial taxa capable of processing complex plant metabolites.^75^ Taken together, these metabolic changes illustrate a dynamic response to xenobiotics, transitioning from acute metabolic stress at 4 h to broader disruptions in fermentation, redox balance, and membrane integrity by 24 h. Additionally, the delayed appearance of heme-related and phytochemical derivatives further suggests changes in microbial composition or functional capacity over time. Overall, the time-dependent metabolic shifts observed in response to EDCs emphasize the relevance of studying different exposure windows to better understand the functional impact of xenobiotic–microbiota interactions.

Although immunological end points were not directly evaluated in this study, several metabolites altered by EDC exposure—including bile acids, lipids, aromatic compounds, porphyrin derivatives, methyl donors, dipeptides and plant-derived metabolites—have been previously linked to immune modulation in other experimental contexts. To facilitate interpretation, these literature-based associations are summarized in Supplementary Table S3 and discussed below. These associations are exploratory and should not be interpreted as evidence of causal immunotoxicity by BPA, TDCPP or PFOA.

Bile acid-related metabolites have well-established immunomodulatory roles. For example, glycodeoxycholic acid has been shown to activate M1 macrophage polarization via the S1PR2-nfkb-NLRP3 axis,^76^ while lithocholylglycine and chenodeoxycholic acid—and its derivatives—modulate FXR- and AHR-dependent pathways, influencing gut immune tone.^77,78^ Conversely, reduced stercobilin abundance may indicate diminished production of microbially derived proinflammatory signals, as stercobilin has been associated with TNFα and IL1β induction in mouse models of metabolic dysfunction.^79^ Aromatic and polyphenol-derived metabolites also have potential immunomodulatory properties. Pentamethoxyflavone, and structurally related flavonoids can inhibit proinflammatory cytokines (e.g., TNFα, IL1β, IL6) via suppression of NFκB and MAPK signaling.^80,81^ The microbial flavanol-derived intermediate 5-valerolactone has been linked to enhanced NK cell activity, CD4+ T cell proliferation and reduced NFκB activation in monocytic cells in vitro.^82,83^ Isonicotinic acid and other pyridine analogues have been associated with anti-inflammatory activity via COX-2 inhibition and antioxidant effects, indirectly shaping inflammatory responses.^84,85^ Microbial lipid metabolism exhibited changes that could influence host-microbiome immune interactions. Altered abundance of ceramides, sphingomyelins and phospholipids intermediates suggest membrane remodeling, which may affect the production and composition of outer membrane vesicles (OMVs). OMVs can modulate TLR2 and TLR4 responses through delivery of microbial-associated molecular patterns.^86,87^ Microbial sphingolipids have been implicated in regulating iNKT cell activity and promoting intestinal immune tolerance through CD1d-dependent pathways.^88^ Methyl-donor-related metabolites, such as choline and betaine, showed compound-and time-dependent alterations. Choline-derived microbial metabolites (TMA/TMAO) influence microbiota composition and IL10-producing T cells.^89,90^ Betaine, which was consistently depleted across exposures, is known to suppresses NFκB and inflammasome activation;^91^ its reduction may therefore suggest diminished anti-inflammatory capacity, although this interpretation remains speculative. Porphyrin-related changes may also have immune relevance. The accumulation protoporphyrin IX and deuteroporphyrin IX at 24 h may indicate the release of damage-associated molecular patterns (DAMPs), which are known to activate TLR4 signaling and promote ROS-driven NLRP3 inflammasome activation.^92,93^ This cascade can lead to macrophage activation and contribute to redox imbalance in the host.^94^ Complementarily, early increases in hypoxanthine and xanthine may indicate enhanced xanthine oxidase-mediated ROS production, a process associated with cytokine induction and oxidative stress.^95,96^ Nucleotide pathway alterations, particularly the depletion of pyrimidines at 24 h, may have implications for local immune dynamics. Pyrimidines are essential for proliferation of epithelial and immune cells, and their depletion can constrain T cell expansion or trigger mitochondrial stress responses.^97,98^ Pyrimidine deficiency can also activate the cGAS-STING-TBK1 signaling axis via release of mitochondrial DNA, promoting type I interferon response.^99^ While speculative in our context, the reduction of pyrimidines might act as a danger signal, contributing to a localized immune activation or microbial-host disequilibrium, particularly when combined with other stress signals such as porphyrin accumulation, redox imbalance, or disruptions in one-carbon metabolism. Peptides and amino acid derivatives also displayed changes consistent with immunomodulatory potential. Pro–Phe—particularly in its cyclic microbial form—have been shown to suppresses proinflammatory cytokines via NFκB inhibition in macrophages,^100,101^ while Pro-hydroxyproline have been linked to ERK/MAPK signaling and integrin activation, both relevant local immune responses and inflammation.^102^ 5-(carbamoylamino) pentanoic acid (citrulline), a key intermediate in arginine metabolism, enhances nitric oxide (NO) production in macrophages and T cells, supporting immune effector function.^103^ While Val–Pro has not been characterized as a standalone immunomodulator, it is a core motif in well-established bioactive peptides (e.g., Val-Pro-Pro) with antihypertensive and indirect immune activities.^104^ Lastly, plant-derived metabolites have been associated with diverse immunomodulatory mechanisms in previous studies. For instance, tomatidine suppresses IL6/JAK/STAT3 signaling and Th2 responses,^105,106^ lupeol modulates macrophage activation depending on context,^107,108^ and pyridoxine (vitamin B6) supports T cell homeostasis and cytokine regulation via MAPK and NFκB pathways.^109,110^ While direct evidence is lacking for 16-deoxyvertaline B, its structural classification as a triterpenoid suggests potential immunoregulatory effects like other phytosterols.^111^ While these literature-based associations highlight plausible links between altered microbial metabolism and immune-related pathways, such interpretations remain hypothetical. Dedicated functional assays will be required to determine whether the metabolic shifts observed here translate into immunological consequences in vivo.

In summary, compound- and time-dependent metabolic shifts observed following EDC exposure reveal substantial disruptions of microbial functional pathways and highlight the sensitivity of gut-associated microbial metabolism to environmental chemicals. Although these findings do not establish causality or define immunological outcomes, they illustrate the complex and dynamic nature of xenobiotic–microbiota interactions and identify several metabolites of potential relevance for host physiology. The inability to detect BPA in the targeted analysis and the absence of clear TDCPP-derived products in the untargeted data emphasize the need for compound-optimized workflows, including refined extraction strategies, targeted MS/MS methods, or suspect screening approaches tailored to specific biotransformation products.

Future studies should extend exposure windows, explore repeated or chronic dosing scenarios, and incorporate epithelial or immune cell coculture systems to determine whether the metabolic perturbations observed here translate into measurable host responses. In addition, studies specifically designed to address interindividual and sex-related differences in microbial responses, as well as integrative approaches combining metabolomics with complementary DNA-based analysis, will be essential to further refine the mechanistic links between chemical exposure, microbial metabolism, and potential impacts on host health.

## 5 Conclusion

This study demonstrates that exposure to selected environmental EDCs elicits distinct, compound- and time-dependent alterations in gut microbial metabolism, affecting key functional pathways including bile acid transformation, SCFA-related processes, lipid remodeling, and nucleotide turnover. These findings highlight the sensitivity of microbial metabolic networks to chemical stressors and underscore the value of integrative microbiome-metabolome approaches for uncovering early functional indicators of xenobiotic impact. More broadly, the results reinforce the importance of considering microbiota-mediated mechanisms when evaluating the potential health effects of environmental contaminants.

## Supplementary Material



## Data Availability

Data is available from the corresponding author upon reasonable request.

## Notes

Supplementary Table S1: list of significantly altered metabolites identified by volcano plot analysis following EDC exposure. Supplementary Figure S1: partial least squares discriminant analysis (PLS-DA) of untargeted metabolomic data. Supplementary Table S2: relative abundance values of selected VIP metabolites identified in heatmap analysis. Supplementary Table S3: literature-based classification of EDC-altered metabolites with potential immunomodulatory functions (PDF)

## References

[1] Ahn C., Jeung E. B. (2023). Endocrine-Disrupting
Chemicals and Disease Endpoints. Int. J. Mol.
Sci..

[2] Liang L. (2022). Immunotoxicity mechanisms of perfluorinated compounds
PFOA and PFOS. Chemosphere.

[3] Tang Y. (2020). Immunotoxicity and neurotoxicity of bisphenol A and microplastics
alone or in combination to a bivalve species, Tegillarca granosa. Environ. Pollut..

[4] Li X., Li N., Rao K., Huang Q., Ma M. (2020). In Vitro Immunotoxicity
of Organophosphate Flame Retardants in Human THP-1-Derived Macrophages. Environ. Sci. Technol..

[5] Ma Y., Liu H., Wu J., Yuan L., Wang Y., Du X., Wang R., Marwa P. W., Petlulu P., Chen X. (2019). The adverse
health effects of bisphenol A and related toxicity mechanisms. Environ. Res..

[6] Post G. B., Cohn P. D., Cooper K. R. (2012). Perfluorooctanoic
acid (PFOA), an
emerging drinking water contaminant: a critical review of recent literature. Environ. Res..

[7] Cho S. H. (2023). Reproductive disorders
linked to the interaction between sex steroid
and thyroid hormonal activities, oxidative stress responses, and the
rate of metabolism of tris (1,3-dichloro-2-propyl) phosphate (TDCPP)
in zebrafish. Ecotoxicol. Environ. Saf..

[8] Teffera M. (2024). Diverse mechanisms by which chemical pollutant exposure
alters gut
microbiota metabolism and inflammation. Environ.
Int..

[9] Cimmino I., Fiory F., Perruolo G., Miele C., Beguinot F., Formisano P., Oriente F. (2020). Potential Mechanisms of Bisphenol
A (BPA) Contributing to Human Disease. Int.
J. Mol. Sci..

[10] Belkaid Y., Hand T. W. (2014). Role of the microbiota in immunity
and inflammation. Cell.

[11] Sommer F., Bäckhed F. (2013). The gut microbiota-masters of host
development and
physiology. Nat. Rev. Microbiol..

[12] Koontz J. M. (2019). The Role of the Human Microbiome in Chemical Toxicity. Int. J. Toxicol..

[13] Calero-Medina L. (2023). Dietary exposure to
endocrine disruptors in gut microbiota: A systematic
review. Sci. Total Environ..

[14] Shreiner A. B., Kao J. Y., Young V. B. (2015). The gut microbiome in health and
in disease. Curr. Opin. Gastroenterol..

[15] Levy M., Kolodziejczyk A. A., Thaiss C. A., Elinav E. (2017). Dysbiosis and the immune
system. Nat. Rev. Immunol..

[16] Fan Y., Pedersen O. (2021). Gut microbiota in human metabolic health and disease. Nat. Rev. Microbiol..

[17] Gálvez-Ontiveros Y., Páez S., Monteagudo C., Rivas A. (2020). Endocrine Disruptors
in Food: Impact on Gut Microbiota and Metabolic Diseases. Nutrients.

[18] Claus S. P., Guillou H., Ellero-Simatos S. (2016). The gut microbiota:
A major player
in the toxicity of environmental pollutants?. npj Biofilms Microbiomes.

[19] Chen X., Zhang Z., Hsueh Y., Zhang C., Yu J., Zhu J., Niu J., Yin N., Zhang J., Cui X. (2025). Interactions between
environmental pollutants and gut microbiota:
A review connecting the conventional heavy metals and the emerging
microplastics. Environ. Res..

[20] Dempsey J. L., Little M., Cui J. Y. (2019). Gut microbiome: An intermediary to
neurotoxicity. Neurotoxicol..

[21] National Academies of Sciences Environmental Chemicals, the Human Microbiome, and Health Risk: A Research Strategy; The National Academies Press, 2018. 10.17226/24960. https://pubmed.ncbi.nlm.nih.gov/29431953/. 29431953

[22] Stevanoska M., Folz J., Beekmann K., Aichinger G. (2024). Physiologically
based kinetic (PBK) modeling as a new approach methodology (NAM) for
predicting systemic levels of gut microbial metabolites. Toxicol. Lett..

[23] Efsa E. F. S. (2016). A Statement
on the Developmental Immunotoxicity of Bisphenol A (BPA): Answer to
the Question from the Dutch Ministry of Health, Welfare and Sport. EFSA J..

[24] Schrenk D., Bignami M., Bodin L., Chipman J. K., del Mazo J., Grasl-Kraupp B., Hogstrand C., Hoogenboom L. R., Leblanc J., EFSA Panel
on Contaminants in the Food Chain
EFSA CONTAM Panel (2020). Risk to Human Health Related to the Presence of Perfluoroalkyl Substances
in Food. EFSA J..

[25] Cao L., Wei L., Du Q., Su Y., Ye S., Liu K. (2023). Spleen Toxicity
of Organophosphorus Flame Retardant TDCPP in Mice and the Related
Mechanisms. Toxics.

[26] Killilea D. W., Chow D., Xiao S. Q., Li C., Stoller M. L. (2017). Flame retardant
tris­(1,3-dichloro-2-propyl)­phosphate (TDCPP) toxicity is attenuated
by N-acetylcysteine in human kidney cells. Toxicol.
Rep..

[27] Feng D., Zhang H., Jiang X., Zou J., Li Q., Mai H., Su D., Ling W., Feng X. (2020). Bisphenol
A exposure
induces gut microbiota dysbiosis and consequent activation of gut-liver
axis leading to hepatic steatosis in CD-1 mice. Environ. Pollut..

[28] Meng L. Y. (2024). Effects of Bisphenol
A and Its Substitute, Bisphenol F, on the Gut
Microbiota in Mice. Biomed. Environ. Sci..

[29] Shi L. (2020). Exposure to Perfluorooctanoic Acid Induces Cognitive
Deficits via
Altering Gut Microbiota Composition, Impairing Intestinal Barrier
Integrity, and Causing Inflammation in Gut and Brain. J. Agric. Food Chem..

[30] Han P., Xue Y., Sun Z., Liu X., Miao L., Yuan M., Wang X. (2025). The toxicological
effects of perfluorooctanoic acid (PFOA) exposure
in large yellow croaker (Larimichthys crocea): exploring the relationship
between liver damage and gut microbiota dysbiosis. Environ. Res..

[31] Sen P. (2024). Exposure to environmental
toxicants is associated with gut microbiome
dysbiosis, insulin resistance and obesity. Environ.
Int..

[32] Wang Q. (2015). Developmental exposure
to the organophosphorus flame retardant tris­(1,3-dichloro-2-propyl)
phosphate: Estrogenic activity, endocrine disruption and reproductive
effects on zebrafish. Aquat. Toxicol..

[33] Huang W., Jin L., Yin H., Tang S., Yu Y., Yang Y. (2023). Assessments
of the effects of tris­(1,3-dichloro-2-propyl) phosphate (TDCPP) on
human intestinal health from the aspects of intestinal flora changes
and cytotoxicity to human cells. Sci. Total
Environ..

[34] Pyambri M., Jaumot J., Bedia C. (2025). Toxicity Assessment
of Organophosphate
Flame Retardants Using New Approach Methodologies. Toxics.

[35] Sha Y. (2024). Chronic exposure to
tris­(1,3-dichloro-2-propyl) phosphate: Effects
on intestinal microbiota and serum metabolism in rats. Ecotoxicol. Environ. Saf..

[36] Qi Y., Yu L., Tian F., Zhao J., Zhai Q. (2023). In vitro models to
study human gut-microbiota interactions: Applications, advances, and
limitations. Microbiol. Res..

[37] Licht T. R., Bahl M. I. (2019). Impact of the gut microbiota on chemical risk assessment. Curr. Opin. Toxicol..

[38] Rodricks J., Huang Y., Mantus E., Shubat P. (2019). Do Interactions Between
Environmental Chemicals and the Human Microbiome Need to Be Considered
in Risk Assessments?. Risk Anal..

[39] Duperron S., Halary S., Gallet A., Marie B. (2020). Microbiome-Aware Ecotoxicology
of Organisms: Relevance, Pitfalls, and Challenges. Front. Public Health.

[40] Tu P. (2020). Gut Microbiome Toxicity:
Connecting the Environment and Gut Microbiome-Associated
Diseases. Toxics.

[41] Dey P. (2020). The role of
gut microbiome in chemical-induced metabolic and toxicological murine
disease models. Life Sci..

[42] Gruszecka-Kosowska A., Ampatzoglou A., Aguilera-Gómez M. (2022). Microbiota analysis
for risk assessment of xenobiotics: cumulative xenobiotic exposure
and impact on human gut microbiota under One Health approach. EFSA J..

[43] Koppel N., Rekdal V. M., Balskus E. P. (2018). Chemical
transformation of xenobiotics
by the human gut microbiota. Sci..

[44] O’Donnell M. M., Rea M. C., Shanahan F., Ross R. P. (2018). The use
of a mini-bioreactor
fermentation system as a reproducible, high-throughput ex vivo batch
model of the distal colon. Front. Microbiol..

[45] Isenring J., Bircher L., Geirnaert A., Lacroix C. (2023). In vitro human gut
microbiota fermentation models: opportunities, challenges, and pitfalls. Microbiome Res. Rep..

[46] Zünd J. N., Plüss S., Mujezinovic D., Menzi C., von Bieberstein P. R., de Wouters T., Lacroix C., Leventhal G. E., Pugin B. (2024). A flexible high-throughput cultivation protocol to assess the response
of individuals’ gut microbiota to diet-drug-and host-related
factors. ISME Commun..

[47] Van
De Steeg E. (2018). An Ex Vivo Fermentation Screening Platform to Study
Drug Metabolism by Human Gut Microbiota. Drug
Metab. Dispos..

[48] Douglas, S. ; Auld, Ph. D. ; Microplate Selection and Recommended Practices in high-throughput Screening and Quantitative Biology; Assay Guidance Manual, 2020. 32520474

[49] Karimi
Douna B., Yousefi H. (2023). Removal of PFAS by Biological Methods. Asian pac. j. environ. cancer.

[50] Panuwet P. (2016). Biological Matrix Effects in Quantitative Tandem Mass
Spectrometry-Based
Analytical Methods: Advancing Biomonitoring. Crit. Rev. Anal. Chem..

[51] Moirana R. L., Kivevele T., Mkunda J., Mtei K., Machunda R. (2021). Trends towards
Effective Analysis of Fluorinated Compounds Using Inductively Coupled
Plasma Mass Spectrometry (ICP-MS). J. Anal.
Methods Chem..

[52] Grgas D., Petrina A., Štefanac T., Bešlo D., Landeka Dragičević T. (2023). A Review:
Per- and Polyfluoroalkyl
Substances—Biological Degradation. Toxics.

[53] Majedi S. M., Lai E. P. C. (2018). Mass Spectrometric Analysis of Bisphenol A Desorption
from Titania Nanoparticles: Ammonium Acetate, Fluoride, Formate, and
Hydroxide as Chemical Desorption Agents. Methods
Protoc..

[54] Dorival-García N., Zafra-Gómez A., Navalón A., Vílchez J. L. (2012). Improved
sample treatment for the determination of bisphenol A and its chlorinated
derivatives in sewage sludge samples by pressurized liquid extraction
and liquid chromatography–tandem mass spectrometry. Talanta.

[55] Wilczewska K., Namies̈nik J., Wasik A. (2015). Troubleshooting of the determination
of bisphenol A at ultra-trace levels by liquid chromatography and
tandem mass spectrometry. Anal. Bioanal. Chem..

[56] Chi L., Tu P., Ru H., Lu K. (2021). Studies of xenobiotic-induced gut
microbiota dysbiosis: from correlation to mechanisms. Gut Microbes.

[57] Hong T. (2023). Bisphenol A induced
hepatic steatosis by disturbing bile acid metabolism
and FXR/TGR5 signaling pathways via remodeling the gut microbiota
in CD-1 mice. Sci. Total Environ..

[58] Kasprzak-Drozd K., Oniszczuk T., Stasiak M., Oniszczuk A. (2021). Beneficial
Effects of Phenolic Compounds on Gut Microbiota and Metabolic Syndrome. Int. J. Mol. Sci..

[59] Kiriyama Y., Tokumaru H., Sadamoto H., Kobayashi S., Nochi H. (2024). Effects of Phenolic Acids Produced
from Food-Derived Flavonoids and
Amino Acids by the Gut Microbiota on Health and Disease. Molecules.

[60] Yang G., Hong S., Yang P., Sun Y., Wang Y., Zhang P., Jiang W., Gu Y. (2021). Discovery
of an ene-reductase
for initiating flavone and flavonol catabolism in gut bacteria. Nat. Commun..

[61] Salim S. Y., Kaplan G. G., Madsen K. L. (2014). Air pollution
effects on the gut
microbiota: A link between exposure and inflammatory disease. Gut Microbes.

[62] Morrison D. J., Preston T. (2016). Formation of short
chain fatty acids by the gut microbiota
and their impact on human metabolism. Gut Microbes.

[63] Rodríguez-Daza M. C. (2021). Polyphenol-Mediated
Gut Microbiota Modulation: Toward Prebiotics
and Further. Front. Nutr..

[64] Rio P., Gasbarrini A., Gambassi G., Cianci R. (2024). Pollutants, microbiota
and immune system: frenemies within the gut. Front. Public Health.

[65] Zubeldia-Varela E., Macías-Camero A., Pérez-Gordo M. (2023). From Bacteria to Host: Deciphering
the Impact of Sphingolipid Metabolism on Food Allergic Reactions. Curr. Treat. Options Allergy.

[66] Mausz M. A. (2022). Microbial uptake dynamics of choline and glycine
betaine in coastal
seawater. Limnol. Oceanogr..

[67] Reed A. D., Fletcher J. R., Huang Y. Y., Thanissery R., Rivera A. J., Parsons R. J., Stewart A. K., Kountz D. J., Shen A., Balskus E. P. (2022). The Stickland Reaction
Precursor trans-4-Hydroxy-l-Proline Differentially Impacts the Metabolism
of Clostridioides difficile and Commensal Clostridia. mSphere.

[68] Gherghina M. E. (2022). Uric Acid and Oxidative Stress—Relationship with Cardiovascular,
Metabolic, and Renal Impairment. Int. J. Mol.
Sci..

[69] Liu X., Ke L., Lei K., Yu Q., Zhang W., Li C., Tian Z. (2023). Antibiotic-induced gut microbiota dysbiosis has a functional
impact
on purine metabolism. BMC Microbiol..

[70] Létoffé S., Heuck G., Delepelaire P., Lange N., Wandersman C. (2009). Bacteria capture
iron from heme by keeping tetrapyrrol skeleton intact. Proc. Natl. Acad. Sci. U. S. A..

[71] Halpern D., Gruss A. (2015). A sensitive bacterial-growth-based test reveals how intestinal Bacteroides
meet their porphyrin requirement. BMC Microbiol..

[72] Wu H.-l., Gong Y., Ji P., Xie Y. f., Jiang Y. Z., Liu G. y. (2022). Targeting nucleotide metabolism:
a promising approach
to enhance cancer immunotherapy. J. Hematol.
Oncol..

[73] Kim S. H. (2025). Altered heme metabolism
and hemoglobin concentration due to empirical
antibiotics-induced gut dysbiosis in preterm infants. Comput. Struct. Biotechnol. J..

[74] Ravishankar S. (2024). Fluoropyrimidines affect de novo pyrimidine
synthesis impairing biofilm
formation in Escherichia coli. Biofilm.

[75] Liou C. S. (2024). Gut microbiota gate host exposure to metabolites from
dietary Solanums. bioRxiv.

[76] Deng M., Liu J., Lou Y., Qiu Y.-Q. (2025). Glycodeoxycholic acid stimulates
antitumor immune response by driving macrophages toward M1 phenotype
in hepatocellular carcinoma. Gut.

[77] Lian S., Lu M., Jiajing L., Zhang B., Fang Y., Wang X., Zheng M., Ni Y., Xu G., Yang Y. (2025). Conjugated Lithocholic
Acid Activates Hepatic TGR5 to Promote Lipotoxicity
and MASLD-MASH Transition by Disrupting Carnitine Biosynthesis. Adv. Sci..

[78] Fling R. R., Zacharewski T. R. (2021). Aryl hydrocarbon receptor (Ahr) activation by 2,3,7,8-tetrachlorodibenzo-p-dioxin
(tcdd) dose-dependently shifts the gut microbiome consistent with
the progression of non-alcoholic fatty liver disease. Int. J. Mol. Sci..

[79] Sanada S., Suzuki T., Nagata A., Hashidume T., Yoshikawa Y., Miyoshi N. (2020). Intestinal microbial
metabolite stercobilin
involvement in the chronic inflammation of ob/ob mice. Sci. Rep..

[80] Yang G. (2018). Nobiletin and 5-Hydroxy-6,7,8,3′,4′-pentamethoxyflavone
Ameliorate 12-O-Tetradecanoylphorbol-13-acetate-Induced Psoriasis-Like
Mouse Skin Lesions by Regulating the Expression of Ki-67 and Proliferating
Cell Nuclear Antigen and the Differentiation of CD4+ T Cells through
Mitogen-Activated Protein Kinase Signaling Pathways. J. Agric. Food Chem..

[81] Peng W., Jin Z., Liu J., Zhang Q., Liu W. (2025). Tangeretin modulates
gut microbiota metabolism and macrophage immunity following fecal
microbiota transplantation in obesity. J. Food
Sci..

[82] Márquez
Campos E., Stehle P., Simon M. C. (2019). Microbial Metabolites
of Flavan-3-Ols and Their Biological Activity. Nutrients.

[83] Lee C. C. (2017). 5-(3′,4′-Dihydroxyphenyl-γ-valerolactone), a
Major Microbial Metabolite of Proanthocyanidin, Attenuates THP-1 Monocyte-Endothelial
Adhesion. Int. J. Mol. Sci..

[84] Digby J. E. (2012). Anti-inflammatory effects of nicotinic acid in human monocytes are
mediated by GPR109A dependent mechanisms. Arterioscler.,
Thromb., Vasc. Biol..

[85] Yaqoob S. (2021). Synthesis of Highly
Potent Anti-Inflammatory Compounds (ROS Inhibitors)
from Isonicotinic Acid. Molecules.

[86] Giordano N. P., Cian M. B., Dalebroux Z. D. (2020). Outer membrane
lipid secretion and
the innate immune response to gram-negative bacteria. Infect. Immun..

[87] Qian D. (2025). Bacterial extracellular vesicles for gut microbiome–host communication
and drug development. Acta Pharm. Sin. B.

[88] Brown E. M., Clardy J., Xavier R. J. (2023). Gut microbiome
lipid metabolism and
its impact on host physiology. Cell Host Microbe.

[89] Liu C. (2022). Choline and butyrate beneficially modulate the gut
microbiome without
affecting atherosclerosis in APOE*3-Leiden.CETP mice. Atherosclerosis.

[90] Eslami M. (2024). The Importance of Gut
Microbiota on Choline Metabolism in Neurodegenerative
Diseases. Biomolecules.

[91] Zhao G., He F., Wu C., Li P., Li N., Deng J., Zhu G., Ren W., Peng Y. (2018). Betaine in inflammation: Mechanistic
aspects and applications. Front. Immunol..

[92] Canesin G., Hejazi S. M., Swanson K. D., Wegiel B. (2020). Heme-Derived
Metabolic
Signals Dictate Immune Responses. Front. Immunol..

[93] Ryter S. W. (2021). Significance
of Heme and Heme Degradation in the Pathogenesis of Acute Lung and
Inflammatory Disorders. Int. J. Mol. Sci..

[94] Larsen R., Gouveia Z., Soares M. P., Gozzelino R. (2012). Heme Cytotoxicity
and the Pathogenesis of Immune-Mediated Inflammatory Diseases. Front. Pharmacol..

[95] Battelli M. G., Polito L., Bortolotti M., Bolognesi A. (2016). Xanthine Oxidoreductase-Derived
Reactive Species: Physiological and Pathological Effects. Oxid. Med. Cell. Longevity.

[96] Pratomo I. P., Noor D. R., Kusmardi K., Rukmana A., Paramita R. I., Erlina L., Fadilah F., Gayatri A., Fitriani M., Purnomo T. T. H. (2021). Xanthine Oxidase-Induced Inflammatory Responses
in Respiratory Epithelial Cells: A Review in Immunopathology of COVID-19. Int. J. Inflammation.

[97] Peeters M. J. W., Aehnlich P., Pizzella A., Mølgaard K., Seremet T., Met Ö., Rasmussen L. J., thor Straten P., Desler C. (2021). Mitochondrial-Linked De Novo Pyrimidine
Biosynthesis Dictates Human T-Cell Proliferation but Not Expression
of Effector Molecules. Front. Immunol..

[98] Fairbanks L. D., Bofill M., Ruckemann K., Simmonds H. A. (1995). Importance of Ribonucleotide
Availability to Proliferating T-lymphocytes from Healthy Humans: DISPROPORTIONATE
EXPANSION OF PYRIMIDINE POOLS AND CONTRASTING EFFECTS OF DE NOVO SYNTHESIS
INHIBITORS. J. Biol. Chem..

[99] Sprenger H. G., MacVicar T., Bahat A., Fiedler K. U., Hermans S., Ehrentraut D., Ried K., Milenkovic D., Bonekamp N., Larsson N. G. (2021). Cellular pyrimidine
imbalance triggers mitochondrial DNA–dependent innate immunity. Nat. Metab..

[100] Kim K. (2015). Cyclo­(Phe-Pro) Produced by the Human Pathogen
Vibrio
vulnificus Inhibits Host Innate Immune Responses through the NF-κB
Pathway. Infect. Immun..

[101] Lee W., Lee S. H., Kim M., Moon J. S., Kim G. W., Jung H. G., Kim I. H., Oh J. E., Jung H. E., Lee H. K. (2018). Vibrio vulnificus quorum-sensing molecule cyclo­(Phe-Pro)
inhibits RIG-I-mediated antiviral innate immunity. Nat. Commun..

[102] Ide K. (2021). The dipeptide prolyl-hydroxyproline
promotes cellular
homeostasis and lamellipodia-driven motility via active β1-integrin
in adult tendon cells. J. Biol. Chem..

[103] Wu G., Bazer F. W., Davis T. A., Kim S. W., Li P., Marc Rhoads J., Carey Satterfield M., Smith S. B., Spencer T. E., Yin Y. (2008). Arginine metabolism and nutrition in growth, health and disease. Amino Acids.

[104] Chakrabarti S., Wu J. (2015). Milk-Derived Tripeptides IPP (Ile-Pro-Pro)
and VPP (Val-Pro-Pro) Promote Adipocyte Differentiation and Inhibit
Inflammation in 3T3-F442A Cells. PLoS One.

[105] Kuo C. Y., Huang W. C., Liou C. J., Chen L. C., Shen J. J., Kuo M. L. (2017). Tomatidine Attenuates
Airway Hyperresponsiveness
and Inflammation by Suppressing Th2 Cytokines in a Mouse Model of
Asthma. Mediators Inflammation.

[106] Parafati M., Shenoy T. S., Thwin Z., Parlavecchio M., Malany S. (2025). Tomatidine Attenuates Inflammatory Responses to Exercise-Like
Stimulation in Donor-derived Skeletal Muscle Myobundles. Med. Res. Arch..

[107] Das A. (2017). Antileishmanial and
immunomodulatory activities of
lupeol, a triterpene compound isolated from Sterculia villosa. Int. J. Antimicrob. Agents.

[108] Dalimunthe A., Carensia Gunawan M., Dhiya Utari Z., Dinata M. R., Halim P., Estherina S., Pakpahan N., Sitohang A. I., Sukarno M. A., Yuandani, Harahap Y. (2024). In-depth
analysis of lupeol: delving into the diverse pharmacological profile. Front. Pharmacol..

[109] Stach K., Stach W., Augoff K. (2021). Vitamin B6
in Health
and Disease. Nutrients.

[110] Du X. (2020). Vitamin
B6 prevents excessive inflammation by reducing
accumulation of sphingosine-1-phosphate in a sphingosine-1-phosphate
lyase–dependent manner. J. Cell. Mol.
Med..

[111] Olatunde O. Z., Yong J., Tian D., Lu C. (2025). Comprehensive
review of plant-derived triterpenoid types, structures and cytotoxicity:
an update from 2015 to 2024. Org. Biomol. Chem..

