# Supplementary material for: Microbiota-Driven Metabolic Alterations Induced by BPA, TDCPP and PFOA in an Ex Vivo Human Fecal Fermentation Model
Source: Chem Res Toxicol. 2026 Mar 17;39(4):646–58. doi: 10.1021/acs.chemrestox.5c00516 (PMC13100943; doi:10.1021/acs.chemrestox.5c00516)
Supplement: Supplementary file 1 [file tx5c00516_si_001.pdf]

Supporting Information

Microbiota-Driven Metabolic Alterations Induced by BPA, TDCPP and PFOA in an *Ex vivo* Human Fecal Fermentation Model

Oscar Sabuz[1], Jacob Folz[5], Deepika Deepika[1,3,4], Jordi Blanco[2], Marta Schuhmacher[1], Georg Aichinger[5], Vikas Kumar*[1,3,4]

[1] *Environmental Engineering Laboratory, TecnATox group, Departament d'Enginyeria Quimica, Universitat Rovira i Virgili, Av. Països Catalans 26, 43007 Tarragona, Catalonia, Spain.*
[2] *Laboratory of Toxicology and Environmental Health, Research in Neurobehavior and Health (NEUROLAB), School of Medicine, IISPV, Universitat Rovira i Virgili, 43201 Reus, Catalonia, Spain.*
[3] *Pere Virgili Health Research Institute (IISPV), Department of Chemical Engineering, Universitat Rovira I Virgili, 43007 Tarragona, Spain*
[4] *German Federal Institute for Risk Assessment (BfR), 10589 Berlin, Germany*
[5] *Laboratory of Toxicology, Department of Health Sciences and Technology, ETH Zürich, 8092 Zurich, Switzerland.*

Corresponding author: Vikas Kumar (vikas.kumar@urv.cat)

Table of Contents

Table S1. Volcano plot-derived list of significantly altered metabolites S2-S7

Figure S1. PLS-DA score plots of untargeted metabolomic data S7

Table S2. Relative abundance values of selected VIP metabolites S8-S9

Table S3. Literature-based classification of EDC-altered metabolites S9-S10

Supplementary Table S1. List of significantly altered metabolites identified by volcano plot analysis following EDC exposure. This table reports all annotated metabolites that were significantly altered ($p<0.05$) in response to BPA, TDCPP or PFOA (75 μM) after 4 or 24h of *ex vivo* fecal fermentation. Values represent the Log2(FC) between exposed and control samples, and the corresponding p-value from unpaired Student's t-tests (n=3). Metabolites are ranked by compound and timepoint. Only features with MS/MS-confirmed annotation and unique entries were included.

**BPA 4h**				
**Name**	**FC**	**Log_2(FC)**	**p**	**-Log_{10}(p)**
Isonicotinic acid	1.2244	0.29213	6.06E-04	3.2174
Hypoxanthine	1.9573	0.96884	1.71E-03	2.7662
Xanthine	1.8466	0.88485	2.54E-03	2.5944
N-Methyl-L-leucine	1.0984	0.13543	2.75E-03	2.5602
3-Amino-4-(4-hydroxyphenyl)butyric Acid	1.176	0.23384	5.13E-03	2.2899
Choline	1.2584	0.33161	6.98E-03	2.1563
5-Benzyl-3-phenyl-2-thioxo-4-imidazolidinone	1.0537	0.075419	9.83E-03	2.0073
Pyridoxine	1.1572	0.21061	1.08E-02	1.9676
Pentamethylene bisacetamide	1.0591	0.082846	0.011184	1.9514
Pipecolic acid	1.1204	0.16399	0.013944	1.8556
3-Aminopyridine	1.1107	0.15142	0.016433	1.7843
Phe-Leu	1.1422	0.19183	2.25E-02	1.6483
1-(2-Carboxyethyl)-1H-imidazole	1.0971	0.13374	0.025411	1.595
Histamine	1.1899	0.2509	0.029613	1.5285
2-Amino-4-methylpentanoic acid ethylamide	1.1125	0.15381	0.032149	1.4928
N-Propylamphetamine	1.804	0.85117	0.039097	1.4079
4-aminovaleric acid betaine	1.0789	0.10955	0.043834	1.3582
Valylphenylalanine	1.0651	0.09102	0.050005	1.301
5-Valerolactone	0.85473	-0.22646	1.15E-04	3.9381
Dimethyl sulfoxide	0.90317	-0.14693	5.99E-04	3.2227
Tangeritin	0.86851	-0.20339	2.81E-03	2.5516
Lithocholylglycine	0.74619	-0.42238	3.14E-03	2.503
4-Cholestenone	0.73085	-0.45235	5.77E-03	2.2386
3-Amino-4-fluoro-N,N-dimethylbenzamide	0.87375	-0.1947	7.11E-03	2.1479
Pro-Phe	0.37639	-1.4097	8.00E-03	2.0967
N-(Eicosanoyl)sphingosine	0.68246	-0.55118	0.012007	1.9206
Glycerol 1-myristate	0.7873	-0.34502	0.025579	1.5921
5,6,7,3',4'-Pentamethoxyflavone	0.91535	-0.1276	0.029064	1.5366

Benzophenone	0.41881	-1.2556	0.042285	1.3738
**BPA 24h**				
**Name**	**FC**	**$Log_2(FC)$**	**p**	**$-Log_{10}(p)$**
16-Deoxyvertaline B	1.9679	0.97667	1.11E-03	2.9553
D-erythro-N-stearoylsphingosine	1.1386	0.18731	1.32E-03	2.878
Sipeimine	1.4182	0.50403	3.34E-03	2.476
Protoporphyrin IX	1.3177	0.39801	5.44E-03	2.2644
N-Oleoylglycine	1.8248	0.86771	5.58E-03	2.2537
Chenodeoxycholic acid	1.8478	0.88584	6.01E-03	2.2212
Ceramide (d18:1/16:0)	1.4108	0.49651	1.33E-02	1.8749
5-Valerolactone	1.4485	0.53452	1.33E-02	1.8746
5-O-Demethylnobiletin	1.1715	0.22831	1.63E-02	1.789
Deuteroporphyrin IX	1.8553	0.89163	0.024801	1.6055
beta-Carotene	1.3176	0.39795	0.027004	1.5686
Lupeol	1.8555	0.89183	0.032064	1.494
2,2,5,7,8-Pentamethyl-6-hydroxychroman	1.4852	0.57061	0.037927	1.421
N-Propylamphetamine	1.3962	0.48147	0.044752	1.3492
Choline	1.2604	0.33385	0.046546	1.3321
1-Palmitoyl-2-oleoyl-sn-glycero-3-phosphoethanolamine	0.59287	-0.7542	7.69E-04	3.1142
Hypoxanthine	0.27605	-1.857	3.95E-03	2.4038
1-Cyclopropyl-6-fluoro-1,2,3-benzotriazole	0.85305	-0.22931	4.46E-03	2.351
5-Benzyl-3-phenyl-2-thioxo-4-imidazolidinone	0.77253	-0.37234	5.63E-03	2.2493
N-Methyl-L-leucine	0.79597	-0.32921	6.79E-03	2.1679
3-Phenyl-beta-alanine	0.76277	-0.39067	7.29E-03	2.1373
Xanthine	0.25629	-1.9641	0.02716	1.5661
Scutellarein tetramethyl ether	0.63311	-0.65946	3.39E-02	1.4701
2-Indolinone	0.76275	-0.39073	0.037012	1.4317
His-Pro	0.8433	-0.24589	0.042829	1.3683
**TDCPP 4h**				
**Name**	**FC**	**$Log_2(FC)$**	**p**	**$-Log_{10}(p)$**
Hypoxanthine	3.2045	1.6801	7.23E-04	3.1411
N-Propylamphetamine	2.7996	1.4852	1.99E-03	2.7016
Xanthine	2.2196	1.1503	2.28E-03	2.6421
Choline	1.4608	0.54679	2.79E-03	2.5538
3-Amino-4-(4-hydroxyphenyl) butyric Acid	1.2683	0.3429	5.10E-03	2.2923
(2S)-2-Amino-2-indan-2-ylacetic acid	1.7188	0.78141	6.49E-03	2.1876
Isonicotinic acid	1.3445	0.42702	7.12E-03	2.1474
L-Tryptophan	1.2045	0.26838	0.012144	1.9156
Val-Pro	1.2996	0.37807	0.012505	1.9029
Leu-Pro	1.4068	0.49245	0.018661	1.7291
Proline-hydroxyproline	1.539	0.62199	0.03965	1.4018
3-Aminopyridine	1.0976	0.13429	0.040044	1.3975

3-Phenyl-beta-alanine	1.0985	0.13558	0.044279	1.3538
Heptadecasphinganine	1.5686	0.64946	0.047579	1.3226
Dimethyl sulfoxide	0.8577	-0.22145	3.30E-05	4.4821
5-(Carbamoylamino)pentanoic acid	0.77667	-0.36462	1.33E-04	3.8768
4-Cholestenone	0.76156	-0.39296	1.55E-03	2.8109
5-Valerolactone	0.4844	-1.0457	2.37E-03	2.6252
Glycerol 1-myristate	0.59504	-0.74895	2.90E-03	2.538
Pentamethylene bisacetamide	0.85345	-0.22862	3.28E-03	2.4847
Lithocholylglycine	0.75275	-0.40975	4.60E-03	2.3373
Betaine	0.89592	-0.15856	0.0081017	2.0914
Ethyl 2-amino-4-hydroxy-5-pyrimidinecarboxylate	0.87248	-0.19681	0.0083497	2.0783
Protoporphyrin IX	0.76289	-0.39045	0.009482	2.0231
Pro-Phe	0.39912	-1.3251	0.0096947	2.0135
Thiabendazole	0.65286	-0.61515	1.01E-02	1.9973
Tetramethylammonium	0.86161	-0.2149	0.011206	1.9506
1-Monolinoleoyl-rac-glycerol	0.82404	-0.27921	0.016544	1.7814
Hexamethylquercetagetin	0.79838	-0.32485	0.020515	1.6879
2-Amino-4-methylpentanoic acid ethylamide	0.87646	-0.19023	0.020865	1.6806
Quinoline-2,8-diol	0.88294	-0.17962	0.022178	1.6541
1-Palmitoyl-2-oleoyl-sn-glycero-3-phosphoethanolamine	0.50206	-0.99408	0.029967	1.5234
Palmitoyl sphingomyelin	0.62804	-0.67107	3.86E-02	1.4132
Ortophosphate	0.87117	-0.19897	0.046721	1.3305
**TDCPP 24h**				
**Name**	**FC**	**Log_2(FC)**	**p**	**-Log_{10}(p)**
5-Valerolactone	5.1609	2.3676	1.21E-03	2.9159
16-Deoxyvertaline B	2.6284	1.3942	1.69E-03	2.771
Deuteroporphyrin IX	3.2793	1.7134	7.03E-03	2.1532
Ceramide (d18:1/16:0)	1.5464	0.62892	1.20E-02	1.9207
Glycodeoxycholic acid	1.4877	0.57307	1.59E-02	1.7975
Chenodeoxycholic acid	2.3395	1.2262	1.65E-02	1.7814
5-O-Demethylnobiletin	1.219	0.28572	1.70E-02	1.7686
Lupeol	2.4552	1.2958	1.89E-02	1.7246
Stercobilin	1.5844	0.66395	0.01897	1.7219
L-Tryptophan	1.4373	0.52333	0.021604	1.6655
Sipeimine	1.3331	0.41484	0.022672	1.6445
Lithocholylglycine	1.8263	0.8689	2.64E-02	1.5783
4-Cholestenone	1.8705	0.90339	0.026754	1.5726
N-Oleoylglycine	1.8556	0.89189	0.034319	1.4645
Isonicotinic acid	1.1593	0.21323	0.040779	1.3896
Hypoxanthine	0.15059	-2.7313	8.38E-05	4.0766
1-Monolinoleoyl-rac-glycerol	0.57295	-0.80351	3.42E-03	2.4654
Xanthine	0.13912	-2.8456	3.51E-03	2.4551
Histamine	0.76972	-0.37759	9.64E-03	2.0157

1-Palmitoyl-2-oleoyl-sn-glycero-3-phosphoethanolamine	0.54836	-0.86682	9.69E-03	2.0138
Ethyl 2-amino-4-hydroxy-5-pyrimidinecarboxylate	0.68069	-0.55493	1.09E-02	1.9639
Tetramethylammonium	0.6432	-0.63666	1.71E-02	1.7662
2,3-Dihydroxypropyl octadecanoate	0.70534	-0.50361	0.020996	1.6779
N-Methyl-L-leucine	0.80836	-0.30693	0.030108	1.5213
Heptadecasphinganine	0.85198	-0.2311	0.037153	1.43
Phenyltrimethylammonium cation	0.88353	-0.17865	0.043193	1.3646
**PFOA 4h**				
**Name**	**FC**	**Log_2(FC)**	**p**	**$-Log_{10}$(p)**
Cyclo(prolyltyrosyl)	1.8106	0.85647	2.62E-04	3.5813
Hypoxanthine	3.3585	1.7478	1.50E-03	2.8233
Xanthine	2.0798	1.0565	0.0026357	2.5791
Isonicotinic acid	1.4436	0.52965	0.0026422	2.578
Choline	1.4651	0.55101	0.0063583	2.1967
Proline-hydroxyproline	1.7155	0.7786	0.013088	1.8831
N-Propylamphetamine	2.3469	1.2307	0.018993	1.7214
Phe-Leu	1.1707	0.2274	0.019588	1.708
3-Amino-4-(4-hydroxyphenyl) butyric Acid	1.2396	0.30984	0.020056	1.6978
(2S)-2-Amino-2-indan-2-ylacetic acid	1.9718	0.97949	0.020144	1.6959
3-(2-Hydroxyethyl) indole	1.3419	0.42431	0.021894	1.6597
Val-Pro	1.3282	0.40946	0.024932	1.6032
L-Tryptophan	1.2368	0.30661	0.033919	1.4696
Ceramide (d18:1/16:0)	1.2481	0.31974	0.035869	1.4453
5-(Carbamoylamino)pentanoic acid	0.72586	-0.46225	3.71E-05	4.4312
5-Valerolactone	0.41474	-1.2697	0.0026335	2.5795
Tetramethylammonium	0.85534	-0.22543	0.0029686	2.5274
Glycerol 1-myristate	0.52655	-0.92536	0.0032829	2.4837
Dimethyl sulfoxide	0.88207	-0.18103	0.0034066	2.4677
(5aR,10aR)-Octahydrodipyrrolo[1,2-a:1',2'-d]pyrazine-5,10-dione	0.75298	-0.40932	0.012084	1.9178
N-Methyl-2-pyrrolidone	0.86396	-0.21096	0.015139	1.8199
4-Picoline	0.73481	-0.44456	0.016321	1.7873
Pro-Phe	0.43943	-1.1863	0.020546	1.6873
4-aminovaleric acid betaine	0.73369	-0.44675	2.15E-02	1.6675
2-(4-Morpholinyl) benzaldehyde	0.54188	-0.88395	0.021548	1.6666
Thiabendazole	0.58192	-0.7811	0.025846	1.5876
N-Methyl-L-leucine	0.85083	-0.23305	0.034627	1.4606
1,2-Diamino-2-methylpropane	0.92097	-0.11877	0.035186	1.4536
Ethyl 2-amino-4-hydroxy-5-pyrimidinecarboxylate	0.86268	-0.2131	0.039499	1.4034
Protoporphyrin IX	0.70423	-0.50589	4.17E-02	1.3801
Stercobilin	0.60438	-0.72647	0.043292	1.3636
Benzophenone	0.42331	-1.2402	0.048088	1.318
**PFOA 24h**				

Name	FC	Log_2(FC)	p	-Log_{10}(p)
(2E)-3-Phenyl-1-(piperidin-1-yl)prop-2-en-1-one	3.9492	1.9816	3.17E-05	4.4984
Lauryldiethanolamine	1.4152	0.50098	8.73E-05	4.059
5-Benzyl-3-phenyl-2-thioxo-4-imidazolidinone	3.0375	1.6029	1.22E-04	3.9131
4-Cholestenone	1.9748	0.98171	2.05E-04	3.6885
Lithocholylglycine	2.2365	1.1612	2.64E-04	3.5784
5-O-Demethylnobiletin	1.3577	0.44114	3.30E-04	3.4819
Chenodeoxycholic acid	2.4521	1.294	4.00E-04	3.3984
16-Deoxyvertaline B	2.2486	1.169	4.15E-04	3.3814
1-Cyclopropyl-6-fluoro-1,2,3-benzotriazole	1.8218	0.86533	6.86E-04	3.1635
N-Palmitoyl-D-sphingosine	2.4485	1.2919	8.36E-04	3.0776
Pyridoxine	2.0169	1.0122	9.23E-04	3.0347
Glycodeoxycholic acid	1.9739	0.98105	0.0015854	2.7999
Ceramide (d18:1/16:0)	2.5671	1.3602	0.0019282	2.7148
Solanine D	2.2608	1.1768	0.0022579	2.6463
Lupeol	2.3771	1.2492	0.0036733	2.4349
Deuteroporphyrin IX	2.7923	1.4815	0.0043021	2.3663
N-Oleoylglycine	2.2179	1.1492	0.0057017	2.244
Stercobilin	2.1817	1.1254	0.0069428	2.1585
Tomatidine	2.5475	1.3491	0.0093082	2.0311
3-Phenyl-beta-alanine	1.6091	0.68626	0.010494	1.979
Protoporphyrin IX	1.6268	0.702	0.01196	1.9223
1,2-Diamino-2-methylpropane	1.3382	0.42027	0.01305	1.8844
Cholestan-3-one, (5.alpha.)-	1.6103	0.6873	0.014173	1.8485
2,2,5,7,8-Pentamethyl-6-hydroxychroman	1.6286	0.7036	0.017691	1.7522
5,6,7,3',4'-Pentamethoxyflavone	1.4325	0.51856	0.019213	1.7164
D-erythro-Sphinganine	1.3262	0.40733	0.021871	1.6601
1-Palmitoyl-sn-glycero-3-phosphocholine	1.4149	0.50073	0.048541	1.3139
Hypoxanthine	0.14075	-2.8288	2.67E-05	4.5741
Choline	0.31328	-1.6745	3.78E-05	4.4223
Xanthine	0.17289	-2.5321	1.02E-04	3.9902
Scutellarein tetramethyl ether	0.66532	-0.58789	3.19E-04	3.4959
Pipecolic acid	0.78036	-0.35778	0.0025194	2.5987
Benzophenone	0.70583	-0.50262	3.50E-03	2.4562
N,N,N-Trimethyl-5-[(tricyclo[3.3.1.1(3,7)]dec-1-ylmethyl)amino]-1-pentanaminium	0.83429	-0.26138	0.0037679	2.4239
1-Methylxanthine	0.67758	-0.56153	0.0045015	2.3466
2,3-Dihydroxypropyl octadecanoate	0.63721	-0.65015	0.0050716	2.2949
Tetramethylammonium	0.25037	-1.9979	0.0052964	2.276
Ethyl 2-amino-4-hydroxy-5-pyrimidinecarboxylate	0.31257	-1.6778	0.0071792	2.1439
4-aminovaleric acid betaine	0.75871	-0.39837	7.41E-03	2.1304

1-Palmitoyl-2-oleoyl-sn-glycero-3-phosphoethanolamine	0.52116	-0.94021	0.0077503	2.1107
Betaine	0.38766	-1.3671	0.010464	1.9803
Stachydrine	0.87914	-0.18583	0.011444	1.9414
Dimethyl sulfoxide	0.78206	-0.35465	0.01275	1.8945
N-Acetyltyramine	0.85555	-0.22508	0.012932	1.8883
Allysine (not validated)	0.82025	-0.28587	0.013903	1.8569
Thiabendazole	0.65298	-0.6149	0.013966	1.8549
His-Pro	0.80657	-0.31014	0.014184	1.8482
Glycerol 1-myristate	0.78703	-0.34551	0.014918	1.8263
N-Methyl-2-pyrrolidone	0.79353	-0.33364	0.020189	1.6949
(7S,7aR)-N-Methylhexahydro-1H-1,6-epoxypyrrolizin-7-amine	0.81895	-0.28815	0.021405	1.6695
4-Picoline	0.76745	-0.38185	0.022873	1.6407
Quinoline-2,8-diol	0.78302	-0.35288	0.025493	1.5936
2-Palmitoyl-rac-glycerol	0.80118	-0.3198	0.026372	1.5789
1-Monolinoleoyl-rac-glycerol	0.62738	-0.67259	0.02941	1.5315
Pyridine	0.82597	-0.27584	0.030316	1.5183
L-Tryptophan	0.61878	-0.6925	0.032603	1.4867
3-(2-Phenoxyethoxy) aniline	0.89798	-0.15525	0.034791	1.4585

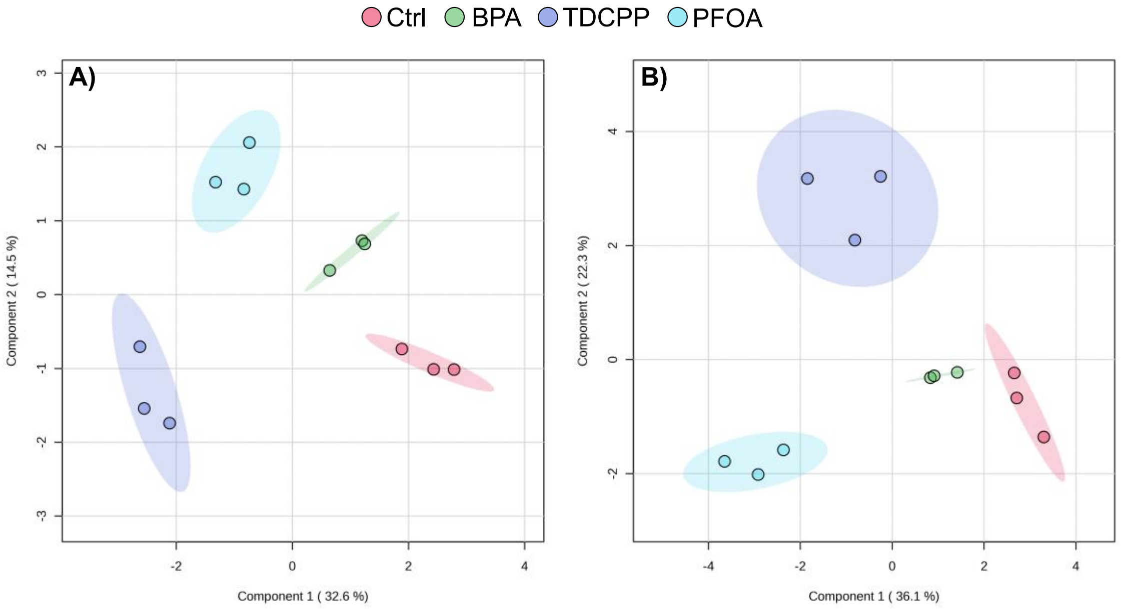

Supplementary Figure S1. Partial Least Squares Discriminant Analysis (PLS-DA) of untargeted metabolomic data. Scores plots show treatment-specific clustering at 4h and 24h after exposure to BPA, TDCPP or PFOA at 75 μM. PLS-DA components reflect the major axes of separation between experimental groups based on normalized metabolite intensities. Each point represents one biological replicate. Ellipses indicate 95% confidence intervals.

Supplementary Table S2. Relative abundance values of selected VIP metabolites identified in heatmap analysis. Normalized relative abundances (mean values) of representative metabolites highlights in the heatmap-based analysis at 4h and 24h. Data are presented as averages from three biological replicates (n=3). Only annotated metabolites with MS/MS confirmation and unique entries were included. Values are shown for control and EDC-treated samples (BPA, TDCPP and PFOA 75 μM).

**Timepoint 4h**				
**Metabolite**	**Ctrl**	**BPA**	**TDCPP**	**PFOA**
Glycodeoxycholic acid	*-0.713*	-0.98	1.07	0.623
Stercobilin	*1.2*	0.359	-0.453	-1.11
Pentamethoxyflavone	*-0.578*	-0.936	1.32	0.19
Isonicotinic acid	-1.36	-0.0791	0.491	0.947
5-valerolactone	*1.03*	0.667	-0.66	-1.03
Glycerol 1-myristate	*1.21*	0.386	-0.582	-1.02
Palmitoyl sphingomyelin	*1.25*	0.294	-1.04	-0.507
Phosphoetanolamines	*1.21*	0.247	-1.18	-0.273
Heptadecaphinganine	*-0.627*	-1.05	1.05	0.628
Choline	-1.38	-0.09	0.734	0.74
Val-pro	*-1.28*	-0.31	0.721	0.867
Pro-Phe	*1.49*	-0.651	-0.515	-0.32
Pro-hydroxyproline	*-1.16*	-0.469	0.577	1.05
5-(carbamoylamino) pentanoic acid	*0.914*	0.791	-0.644	-1.06
Hypoxanthine	*-1.36*	-0.15	0.719	0.788
Xanthine	*-1.47*	0.22	0.711	0.537
Tangeritin	*-0.425*	-1.01	1.34	0.098
Protoporphyrin IX	*1.2*	0.468	-0.526	-1.09
Deuteroporphyrin IX	1.2	0.447	-0.742	-0.903
**Timepoint 24h**				
**Metabolite**	**Ctrl**	**BPA**	**TDCPP**	**PFOA**
Glycodeoxycholic acid	-1.23	-0.15	0.187	1.19
Stercobilin	-1.04	-0.523	0.314	1.25
Chenodeoxycholic acid	-1.43	0.073	0.577	0.782
Lithocholylgycine	-1.22	-0.325	0.449	1.1
Ceramide d18:1/16:0	-1.12	-0.209	0.0181	1.31
N-palmitoyl-D-sphingosine	-1	-0.349	-0.0141	1.37
N-(eicosanoyl)sphingosine	-1.15	-0.337	0.261	1.22
N-oleoglycine	-1.45	0.3	0.29	0.85
Phosphoetanolamines	1.47	-0.234	-0.535	-0.703
Choline	0.457	0.848	0.13	-1.43
Betaine	0.718	0.348	0.415	-1.46
Pyrimidine	0.728	0.654	0.0463	-1.43
Deuteroprophyrin IX	-1.35	-0.148	0.876	0.622
Hypoxanthine	1.42	-0.0306	-0.664	-0.729
Xanthine	1.45	-0.199	-0.796	-0.459
Tomatidine	-1.28	-0.135	0.302	1.12
Solanine D	-1.16	-0.173	0.0648	1.27
Lupeol	-1.43	0.0563	0.689	0.69

16-deoxyvertaline B	-1.45	0.158	0.809	0.479
Pyridoxine	-0.476	-0.532	-0.491	1.5

Supplementary Table S3. Literature-based classification of EDC-altered metabolites with potential immunomodulatory functions. This table compiles selected metabolites identified as significantly altered in response to EDC exposure in the present study (BPA, TDCPP and PFOA) and summarizes their known or putative involvement in immune-related pathways, based on previous literature.

Metabolite	Putative Immune-Related Pathway(s)	Immunological Function	References
**Bile acids**			
Glycodeoxycholic acid	S1PR2, NFκB, NLRP3	M1 macrophage polarization, anti-tumor activity	76
Chenodeoxycholic acid	FXR, AHR	Regulation of gut immunity	77, 78
Lithocholylgycine			
Stercobilin	TNFα, IL1β	Control microbial inflammatory activity	79
**Polyphenol/Aromatic compounds**			
Pentamethoxyflavone	NFκB, MAPK	Inhibition of proinflammatory cytokines	80, 81
Tangeritin			
Isonicotinic acid	COX-2	Anti-inflammatory activity, inhibition of antioxidant effects	82, 83
5-valerolactone	NFκB	Enhance NK cell and CD4+ T cell activity	84, 85
**Lipids**			
Microbial lipids	TLR2, TLR4	Cytokine production	86, 87
Microbial sphingolipids	CD1d, TCR	Modulation of iNKT cells, promotion of immune tolerance	88
**Methyl donors**			
Choline	IL10	Regulation of $IL10^{+}$ T cells	89, 90
Betaine	NFκB, NLRP3, TNFα, iNOX, COX-2	Modulation of inflammatory capacity	91
**Amino acid metabolites**			

Pro-Phe	NFκB	Suppression of proinflammatory cytokine production in macrophages	100, 101
Pro-hydroxyproline	ERK, MAPK, integrins	Regulation of local immune responses	102
Citrulline	NOS	Nitric oxide production, immune effector function	103
**Nucleotide and heme derivatives**			
Pyrimidine	cGAS, STRING, TBK1, IFN-I	Innate immune activation, reduction of T cell expansion	97, 98, 99
Porphyrins	TLR4, NLRP3, ROS	Macrophage activation and redox imbalance	92, 93, 94
**Purines**			
Hypoxanthine	XO, ROS	Cytokine regulation	95, 96
Xanthine			
**Plant-derived compounds**			
Tomatidine	IL6, JAK, STAT3	Suppression of Th2 responses	105, 106
Lupeol	NFκB, NRF2	M2 macrophage polarization, suppression of proinflammatory cytokines, promotes tolerance	107, 108
Pyridoxine	MAPK, NFκB	T cell homeostasis and cytokine regulation	109, 110
